# Using surveillance data for early warning modelling of highly pathogenic avian influenza in Europe reveals a seasonal shift in transmission, 2016–2022

**DOI:** 10.1038/s41598-023-42660-7

**Published:** 2023-09-16

**Authors:** Lene Jung Kjær, Michael P. Ward, Anette Ella Boklund, Lars Erik Larsen, Charlotte Kristiane Hjulsager, Carsten Thure Kirkeby

**Affiliations:** 1 Department of Veterinary and Animal Sciences, Faculty of Health and Medical Sciences, University of Copenhagen, Copenhagen, Denmark; 2 Faculty of Science, Sydney School of Veterinary Science, University of Sydney, Camden, NSW Australia; 3 Statens Serum Institut, Copenhagen, Denmark

**Keywords:** Biological models, Animal disease models

## Abstract

Avian influenza in wild birds and poultry flocks constitutes a problem for animal welfare, food security and public health. In recent years there have been increasing numbers of outbreaks in Europe, with many poultry flocks culled after being infected with highly pathogenic avian influenza (HPAI). Continuous monitoring is crucial to enable timely implementation of control to prevent HPAI spread from wild birds to poultry and between poultry flocks within a country. We here utilize readily available public surveillance data and time-series models to predict HPAI detections within European countries and show a seasonal shift that happened during 2021–2022. The output is models capable of monitoring the weekly risk of HPAI outbreaks, to support decision making.

## Introduction

In recent years, occurrences of highly pathogenic avian influenza (HPAI) in domestic and wild birds have increased globally, posing a threat to animal welfare and public health^1–4^ as well as causing great economic losses when farmed birds become infected^5,6^. Some studies have found evidence of intercontinental spread of avian influenza virus (AIV) from Eurasia to Africa^7^ and North America^8,9^. Transmission from wild to domestic birds is a risk for many poultry farmers, especially regarding HPAI. However, low pathogenic avian influenza (LPAI) can also be transmitted from wild birds to captive birds and poultry, and certain LPAI subtypes (H5 and H7) have the potential to mutate into HPAI in poultry^10^.

In Europe, detections of LPAI and HPAI have been characterized by seasonal oscillations and an increased number of detections during the period November to May^11^. However, during the 2021 and 2022 seasons, detections were also observed during the summer months^12,13^. HPAI causes high mortality, approaching 100%^14^, and infected flocks are culled for welfare reasons and to prevent spread to other flocks^15,16^. Preventive actions to control avian influenza worldwide include reinforcement of biosecurity measures^17^, movement restrictions and vaccination^13,18–20^. Furthermore, biosecurity recommendations (such as indoor housing) are encouraged to prevent infection from wild birds^21^. These measures are enforced during periods with high risk of HPAI virus transmission and in restriction zones around affected farms^22^. Ideally, to predict high-risk periods and areas and to increase the effect of preventive measures, an early warning system should be developed and implemented.

As wild birds cannot be contained in the same way as domestic birds during disease outbreaks, to prevent disease spread it is important to quickly detect AIV in wild bird species and mitigate potential transmission to domestic birds^23^. This can be done using early warning systems, although in the case of AIV challenges include the range of hosts and virus mutations^23^. Early warning systems can be applied to both wild and domestic bird species but are invariably dependent on surveillance data. AIV surveillance within poultry flocks can include the use of diagnostic testing schemes^24^, sentinel birds^25^, surveillance of flock mortality^26^ and morbidity and monitoring the daily feed and water intake and egg production^27^. Early warning systems for AIV in large areas (e.g., country level) can include active and passive surveillance of poultry flocks and wild birds^6^, environmental sampling^28^, complex systems such as hybrid knowledge-based algorithms^29^, and models based on movement data^30,31^. However, there is a need for systems that cover larger study areas beyond national boundaries, incorporate multiple wild- and domestic bird species and use real-time outbreak data. Thus, any system could benefit from the utilization of already available registered information on the occurrence of AIV in neighbouring countries, to predict the risk of transmission in specific countries.

The World Organisation for Animal Health’s World Animal Health Information System (WOAH-WAHIS^32^) platform provides free access to world animal health data. The platform reports surveillance data from veterinary services in WOAH member and non-member countries and territories on WOAH-listed diseases in wildlife and domestic animals. The WAHIS database is publicly accessible and contains data as far back as 2005^32^. Used in a statistical model, these data could facilitate the creation of an early warning system, when combined with a time-series modelling framework such as that proposed by Meyer et al.^33^. This modelling framework has previously been applied in human health modelling^34–37^, as well as modelling rabies in foxes^38^, *Campylobacter* in poultry^39^, and diseases in pigs^40^. Thus, this modelling framework has yet unexplored potential for use in modelling the risk of AIV transmission. In this study, we used registered WOAH-WAHIS data on HPAI in Europe to fit endemic-epidemic time-series models^33^, and evaluated the potential of such models based on accessible data as an early warning system. Our aim was to evaluate the use of WOAH-WAHIS data and the modelling framework to predict detections of HPAI virus on a weekly basis. The objective was to construct an early warning model which can inform decision makers of the risk of outbreaks for the purpose of timely implementation of preventive measures.

## Materials and methods

### Data

We obtained data from the World Organisation for Animal Health (WOAH), where data are continuously made available^32^. As the data included all reported animal diseases, we used the definitions “High pathogenicity avian influenza viruses (poultry) (Inf. with)” and “Influenza A viruses of high pathogenicity (Inf. with) (non-poultry including wild birds)” to select only HPAI outbreaks and detections. Some of the reported events within these categories affected species other than birds and were thus excluded. For simplicity, we defined each data entry as outbreak detection (where outbreak detection includes both outbreaks in domestic birds and detections in wild birds), referring to one or more HPAI detections at the same locality at the same time. Thus, we did not distinguish between domestic and wild birds in our data. Although each entry in the WOAH-WAHIS data reported the number of affected birds, we chose to only model outbreak detections, because some countries (for example Denmark) will not sample and test all wild birds from the same area if it is a known outbreak area (and this could therefore greatly bias the data). Furthermore, domestic birds are usually affected in greater numbers than wild birds and this might skew outbreak severity patterns (if wild and domestic birds are modelled together).

We restricted our analyses on European data to countries that reported HPAI detection data during the study period. We restricted our study region to Europe, and in order to focus on recent years to understand the resurgence of HPAI cases in Europe, and to avoid large temporal gaps in the HPAI data (for example, Denmark had no reported detections between 2006 and 2016), we restricted the study period to be from 1 January 2016 to 5 December 2022. European countries with no reports of HPAI between 2016 and 2022 (e.g., Andorra, Belarus, Kosovo, Liechtenstein, Malta, Monaco, San Marino, and Vatican City) were omitted from our analyses.

We aggregated number of outbreak detections by week (n = 361) and country (n = 37), starting with week 1 in 2016 and ending with week 49 in 2022. For each country, if no outbreak detections were reported in a given week within the study period, we included this week as a “zero detection” observation.

Due to the change in seasonal oscillations observed in Europe during 2021 and particularly 2022 compared to previous years^12,13^, we split our data and ran analyses using only data from 2016 to 2021 (week 1 in 2016 to week 52 in 2021), and models using only data from the last quarter of 2021 and all of 2022 (week 39 in 2021 to week 49 in 2022). We refer to these two data sets as HPAI1621 and HPAI2122. The two datasets overlap in time, as we wanted to include the latest HPAI season in the 2021–2022 dataset, but only keeping week 1–38 for 2021 in the HPAI1621 dataset caused model calibration problems, due to insufficient amount of detection data.

We decided to aggregate the data to weekly HPAI detections, because aggregating over longer periods can mask causality and seasonality in the detections. However, the modelling framework described in this study can easily be adapted to aggregations over longer time periods, the data preparations just need to be adjusted accordingly. As a form of sensitivity analysis, we ran our statistical models with data aggregated bi-weekly, to assess whether the resulting models differed greatly from models with data aggregated weekly (see Sect. "Statistical analysis").

### Landscape variables

Wild birds are natural reservoirs of AIV^41^, so we explored landscape variables that might be associated with presence of wild birds, i.e., coastal areas, waterways, and wetlands, where migratory birds are known to aggregate in high numbers^42^. For each country, we obtained the length of coastline (km) through the CIA World Factbook^43^ and used Global Lakes and Wetlands Database rasters^44^, 1 km^2^ resolution). We used R 4.1.2^45^ package sp^46,47^ to calculate the area (km^2^) of waterways/wetlands within each country. We combined waterways and wetlands and used the following landcover definitions in the raster file to calculate the area within each country: “Lake”, “Reservoir”, “River”, “Freshwater Marsh, Floodplain”, “Coastal Wetland (incl. Mangrove, Estuary, Delta, Lagoon)”, “Pan, Brackish/Saline Wetland”, “Bog, Fen, Mire (Peatland)”, and “Intermittent Wetland/Lake”.

### Statistical analysis

We used the multivariate time-series modelling framework described by Meyer et al.^33^ to model the spatiotemporal relationship between HPAI detections. This modelling framework allows for additive decomposition of time series data into endemic and epidemic components. The HPAI data (outcome variable) was modelled as weekly counts conditional on past observations, following a negative binomial distribution, with the conditional mean decomposing into a random endemic part that incorporates a trend parameter, and an epidemic component composed of autoregressive effects (within-country effects) and neighbourhood effects (between-country effects). The epidemic component captures the occasional (to some extent local) detections while the endemic component explains the baseline rate of detections, usually with a temporal pattern. This endemic component translates, in our case, to the underlying risk of occurrence from factors not included in the model, i.e., transmission from migratory birds coming from countries not included in our study and unreported HPAI virus circulating within the countries included. The main formula for the conditional mean of the negative binomial distribution model is:

$${\mu }_{it}={e}_{it}{\nu }_{it}+{\lambda }_{it}{Y}_{i,t-1 }+{\phi }_{it}\sum_{j\ne i}{w}_{ji}{Y}_{j, t-1}$$

where ${\mu }_{it}$ is the conditional mean per country, *i* and time, *t* (week). The number of expected counts, *e*_*it*_, is modelled as an offset for the endemic log-linear predictor, *ν*_*it*_. The epidemic component is observation-driven and contains a within-country effect (*λ* per country *i* at time *t*), and *Y* denotes the HPAI data, in country *i* at time *t − 1*. The between-country effects (*ϕ*, disease from other regions *j*) are captured by *w*_*ji*_ denoting the spatial weights matrix for the adjacency order of the different countries *o*_*ji*_ (See Supplementary Material for description of the adjacency matrix and Fig. S1) at time *t-1*. It is also possible to include data on infections originating from non-neighbouring countries by incorporating long-range transmission. This can be done by estimating the spatial weights *w*_*ji*_ as a power-law model with distance-decay, *w*_*ij*_ = ${o}_{ji}^{-d}$^33^. Thus, the model incorporates an endemic transmission component (*ν*_*it*_), an epidemic transmission component that splits into within-country transmission effects (*λ*_*it*_), and between-country transmission effects (*ϕ*_it_). All elements within the two components − *ν*_*it*_*, λ*_*it*_*,* and *ϕ*_it_—allow for log-linear predictors as the above equation becomes a rich regression model. For more detailed description of the underlying modelling equations see Meyer et al.^33^, Held et al.^48^, and Paul and Held^49^.

**Figure 1:**
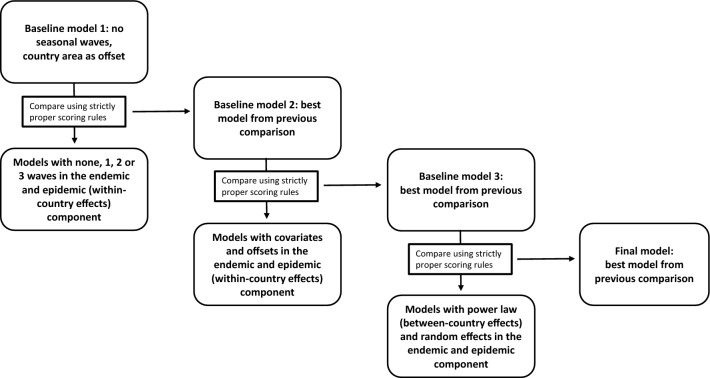
An overview of the different model comparisons and different baseline models used for selecting the final multivariate highly pathogenic avian influenza virus time-series models for the HPAI1621 and HPAI2122 data. Within-country/between-country effects denote that covariates/offsets/seasonality/power law was only included in the within-country/between-country effects of the epidemic component. All models included country area relative to all the country areas as offset in the endemic component.

We used the same model selection process for both the HPAI1621 and HPAI2122 data sets, and Fig. 1 gives an overview of the different model comparisons and selection of final models. We used the package surveillance^33,50^ in R 4.1.2^45^ to fit our models and explored several models with varying structure as described below and in Fig. 1. We divided both our HPAI1621 and HPAI2122 datasets into training sets (week 1 in 2016 to week 46 in 2021 and week 39 in 2021 to week 43 in 2022, respectively), and test sets (week 47 to week 52 in 2021 and week 44 to week 49 in 2022, respectively). We used the respective training data sets to train all our models and the test data sets to check model performance and to compare different model specifications. For both HPAI1621 and HPAI2122 datasets, we first specified a basic model (Baseline model 1 in Fig. 1) with no covariates and no seasonality but with country area relative to all countries (*area_frac*) as offset in the endemic component and simple epidemic between-country effects (only disease transmission from countries directly sharing a border, *w*_*ji*_ = 1(*j* ∼ *i*) = 1(*o*_*ji*_ = 1)):

$${\mu }_{it}={area\_frac}_{i}{\nu }_{i}+\lambda {Y}_{i,t-1 }+\phi \sum_{j\ne i}{w}_{ji}{Y}_{j, t-1},$$

In this basic model, we assumed that both the endemic and epidemic parameters were homogeneous across countries and constant over time, *ν* = exp(*α*^(*ν*)^), *λ* = exp(*α*^(*λ*)^) and *ϕ* = exp(*α*^(*ϕ*)^). For the epidemic components, we used an offset of 1 and for all fixed-effects models, we used the default quasi-Newton penalisation for log-likelihood to maximize the marginal likelihood regarding the variance parameters^37^. We then compared the basic models to other models incorporating 1, 2 and 3 seasonal waves in both the endemic and epidemic components. We chose only to add seasonal waves to the within-country effects in the epidemic components (and not to the between-country effects) to keep model complexity as simple as possible (model equations below depict 3 seasonal waves (*s*)):

$${\mathrm{log}(\nu }_{it})={\alpha }^{\left(\nu \right)}+{\sum }_{s=3}^{s}\left\{{\gamma }_{s}^{(\nu )}\mathrm{sin}\left({\omega }_{s}t\right)+ {\delta }_{s}^{(\nu )}\mathrm{cos}({\omega }_{s}t)\right\}, {\omega }_{1}=\frac{2\pi }{52},{\omega }_{2}=\frac{4\pi }{52}, {\omega }_{3}=\frac{6\pi }{52}$$

$${\mathrm{log}(\lambda }_{it})={\alpha }^{\left(\lambda \right)}+{\sum }_{s=3}^{s}\left\{{\gamma }_{s}^{(\lambda )}\mathrm{sin}\left({\omega }_{s}t\right)+ {\delta }_{s}^{(\lambda )}\mathrm{cos}({\omega }_{s}t)\right\}, {\omega }_{1}=\frac{2\pi }{52},{\omega }_{2}=\frac{4\pi }{52}, {\omega }_{3}=\frac{6\pi }{52}$$

As recommended by Paul and Held^49^, we used strictly proper scoring rules when comparing and validating models. We specifically used the ranked probability score (RPS) and the logarithmic score (logS), and the scores were based on successive one-step-ahead predictions (one-week-ahead forecasts) for the models using the test sets (previously unseen by the models), where we refitted the models up to week *t* to obtain predictions for week *t* + 1. Strictly proper scoring rules provide summary measures by evaluating probabilistic forecasts based on the predictive distribution and the actual count data^51^. The most commonly used strictly proper scoring rules are the logS score and RPS. LogS (calculated as minus the log of the probability estimate for the actual outcome) is highly sensitive to extreme values, as it penalizes low probability events. In contrast RPS generalizes the absolute error and thus measures how well probabilistic forecasts match the observed outcome^49,52^. RPS is less sensitive to extreme values and adds weights to unusually high observed or predicted counts^49,52^. When comparing logS and RPS for different models, we calculate mean scores over a set of predictions. Assessment can be done by simply ordering the mean scores or using permutation tests to test for significant differences^49^. If RPS and logS differed in scoring the best model, we chose to use logS as it is more sensitive to a misprediction in an outbreak period than RPS. For both RPS and logS, a lower score indicates a better mode^49^. We used permutation tests for paired data to test for differences between mean model scores of the two models with the lowest scores^49^ (Fig. 1). The best performing models were then selected as the new baseline models (Fig. 1). We checked if all models were well-calibrated by using a calibration test to assess whether the predictive distribution covered the observed value^52^, and only well-calibrated models were considered when selecting the best performing models. After selecting the best models comparing seasonal waves, we used these as new baseline models (Baseline model 2 in Fig. 1) and added area of waterways/wetlands and length of coastline as log-transformed covariates (*τ*^(*ν*)^(log(*coast* + 1) + log(*wetlands* + 1)), *τ*^(*λ*)^ (log(*coast* + 1) + log(*wetlands* + 1)) or offsets (multiplying log(*coast* + 1) + log(*wetlands* + 1) with *ν*_*i*_ and/or *λ*_*it*_) to the endemic and epidemic components (only for the epidemic within-country effects as these covariates were country-specific) and compared and validated these models to the new baseline models, resulting in selection of another new baseline model for each of the HPAI1621 and HPAI2122 data sets (Baseline model 3 in Fig. 1). Lastly, we compared the latest new baseline models to models with spatial weights as a power-law model with distance-decay, *w*_*ij*_ = ${o}_{ji}^{-d}$ and to models with country-specific (both uncorrelated and correlated) random effects, ${\alpha }_{i}^{(\nu )}\sim N({\alpha }^{(\nu )},{\sigma }_{\nu }^{2})$, ${\alpha }_{i}^{(\lambda )}\sim N({\alpha }^{(\lambda )},{\sigma }_{\lambda }^{2})$, ${\alpha }_{i}^{(\phi )}\sim N({\alpha }^{(\phi )},{\sigma }_{\phi }^{2})$ (assumed to be normally distributed with mean 0 and positive definitive covariance matrix) to capture any remaining heterogeneity not explained by covariates or seasonal waves (for example due to underreporting of outbreaks in certain countries). These random effects were applied to both the endemic and epidemic components of the models (in the latter both for within- and between-country effects), to absorb effects such as country-specific likelihood of detection and occurrence unrelated to landscape factors. As with previous model comparisons, we checked for model calibration and used RPS and logS to select the final best models for forecasting. For models including random effects, we used the Nelder-Mead penalisation for log-likelihood to maximize the marginal likelihood regarding the variance parameters^49^. We used the final best models to run 500 simulations of long-term forecasts to gauge the overall ability of the models to simulate HPAI detections. For the HPAI1621 dataset, we simulated the years 2020–2021 (week 1 in 2020 to week 52 in 2021), whereas for the HPAI2122 dataset we simulated week 40 in 2021 to week 49 in 2022. Forecasting consists of sequential calls to the negative binomial distributions developed in the final models. At each time point (here week number), the mean is determined by using the parameter estimates and the counts simulated at the previous time point. For example, for the first week in 2020 (HPAI1621 dataset), we needed parameter estimates and counts simulated before 2020 and we specified the initial vector of counts used for this forecast as the last week of 2019. For the HPAI2122 models, we used week 39 in 2021.

As a sensitivity analysis, to test if our model results were sensitive to the timescale of our data, we ran models using the HPAI1621 dataset aggregated bi-weekly and compared the final resulting model, its variables, offsets, and coefficients to the final model based on data aggregated weekly. We used the same methods as described above.

## Results

### Data

The data obtained from WOAH-WAHIS^32^ contained 15,595 European HPAI detections during 361 weeks from 37 countries (including Faroe Islands, see Table 1) that reported HPAI data between 1 January 2016 and 5 December 2022. Of these, 8873 were detections in wild birds, whereas 6722 were detections in domestic birds. Table 1 gives a detailed overview of the countries included in our study, the number of wild and domestic detections and the HPAI subtypes reported for each country between 2016 and 2022. Supplementary Table S1 shows the reported detections by subtype and the number of countries that detected the specific subtypes.

**Table 1:** The 37 European countries included in this study, number of detections in wild and domestic birds, highly pathogenic avian influenza subtypes, and number of weeks with and without reported outbreaks for each country between 2016 and 2022 (1 January 2016, to 5 December 2022).

Country*^,‡^	Wild/domestic bird detections	Subtypes reported	Weeks with detections/ weeks with no or missing detections
Albania	2/17	H5N1, H5N8	5/356
Austria	109/10	H5, H5N1, H5N5, H5N8	36/325
Belgium	240/60	H5, H5N1, H5N5, H5N8	73/288
Bosnia & Herzegovina	3/1	H5, H5N1, H5N8	44/317
Bulgaria	16/162	H5, H5N8, “Not specified”	4/357
Croatia	29/19	H5N1, H5N5, H5N8	20/341
Czechia	84/163	H5N1, H5N5, H5N8	47/314
Denmark	569/43	H5, H5N1, H5N3, H5N5, H5N6, H5N8	128/233
Estonia	50/6	H5N1, H5N8	102/259
Faroe Islands†	24/3	H5N1	44/317
Finland	122/2	H5, H5N1, H5N5, H5N6, H5N8	24/337
France	436/2679	H5, H5N1, H5N2, H5N3, H5N8, H5N9	66/295
Germany	2066/565	H5, H5N1, H5N2, H5N3, H5N4, H5N5, H5N6, H5N8	114/247
Greece	30/6	H5N1, H5N5, H5N6, H5N8	11/350
Hungary	106/993	H5N1, H5N5, H5N8	100/261
Iceland	27/1	H5N1	17/344
Ireland	182/28	H5N1, H5N3, H5N6, H5N8	24/337
Italy	131/458	H5N1, H5N5, H5N8, H7N7	77/284
Latvia	24/0	H5N1, H5N8	50/311
Lithuania	20/55	H5N1, H5N8, H7N7	13/348
Luxembourg	7/5	H5N1, H5N8	79/282
Moldova	0/11	H5N1	21/340
Montenegro	2/0	H5N1, H5N5	6/355
Netherlands	742/117	H5, H5N1, H5N3, H5N4, H5N5, H5N6, H5N8	8/353
North Macedonia	10/2	H5, H5N1, H5N8	5/356
Norway	97/8	H5N1, H5N5, H5N8	5/356
Poland	220/491	H5N1, H5N2, H5N5, H5N8	2/359
Portugal	14/34	H5N1, H5N8	108/253
Romania	123/62	H5, H5N1, H5N5, H5N8	51/310
Serbia	50/10	H5N1, H5N2, H5N5, H5N8	84/277
Slovakia	82/19	H5, H5N1, H5N5, H5N6, H5N8	20/341
Slovenia	70/1	H5N1, H5N5, H5N8	39/322
Spain	196/137	H5N1, H5N8	33/328
Sweden	234/51	H5, H5N1, H5N4, H5N5, H5N6, H5N8	27/334
Switzerland	124/4	H5N1, H5N4, H5N6, H5N8	22/339
Ukraine	8/26	H5, H5N8	93/268
United Kingdom	2624/473	H5N1, H5N3, H5N5, H5N6, H5N8	20/341
Total: 37 countries	15,595		

*European countries omitted due to no reported HPAI detections during the study period: Andorra, Belarus, Kosovo, Liechtenstein, Malta, Monaco, San Marino, and Vatican City.

^‡^European countries omitted due to either size and/or being transcontinental: Russia, Turkey.

^†^Although the Faroe Islands belong to Denmark, they have here been treated separately due to the large distance to Denmark.

**Figure 2:**
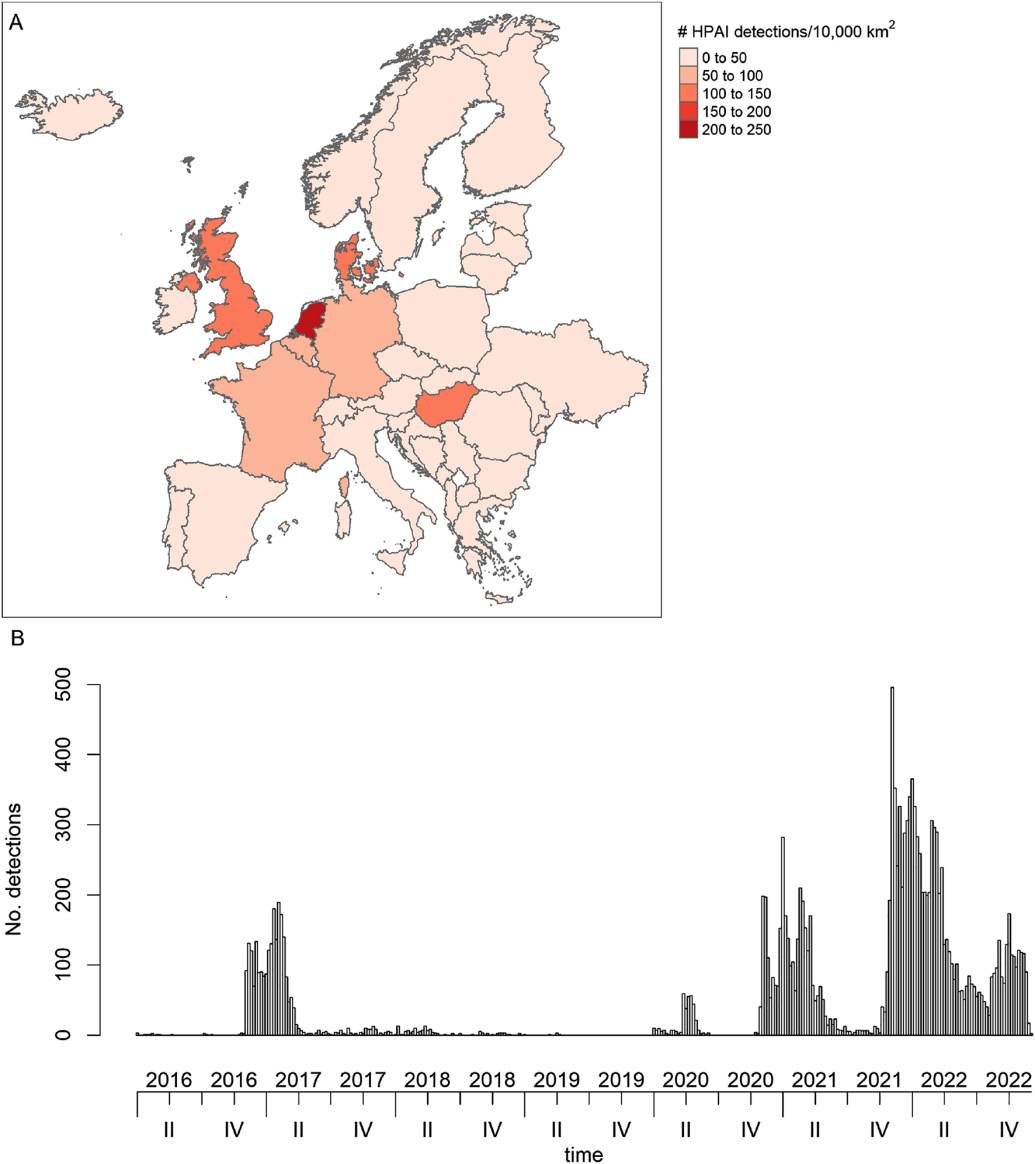
Number of reported highly pathogenic avian influenza (H5 subtype) detections between 2016 and 2022 summed over 37 European countries (including Faroe Islands) shown (**A**) geographically as numbers per 10,000 km^2^, and (**B**) over time. The dataset contained a total of 15,549 detections of varying sizes in both wild and domestic birds. The map in A) was created using the package tmap^64^ in R 4.1.2^45^.

Due to few records of H7 cases, we decided to only use H5 subtypes in our study and removed 46 entries (including 43 entries where subtype was not specified, Supplementary Table S1). This produced a dataset containing 15,549 detections. Due to data constraints, we combined the different H5 subtypes in our analyses (where 60.8% of the detections were H5N1 and 35.7% were H5N8, Supplementary Table S1). Considering H5 subtypes only, France, Germany and the UK were among the countries with the highest number of detections, followed by Hungary, Poland, Italy, the Netherlands, and Denmark. However, when correcting for country size, the highest number of detections per 10,000 km^2^ were found in the Netherlands, followed by Denmark, the UK, Hungary, Germany, Belgium, and France (Fig. 2A). Overall, detections were predominantly reported in the autumn and spring, and large epidemic waves were observed in 2016/2017, 2020, 2021 and 2022 (Fig. 2B), with similar patterns for both wild and domestic birds (Supplementary Fig. S2).

Due to changes in the seasonality of the detections observed in 2021 and particularly in 2022 (Fig. 2B)^12,13^, we created two datasets from our data, before analysing and modelling spatiotemporal relationships. The first data set, hereafter referred to as HPAI1621, contained data from 2016 to 2021 (week 1 in 2016 to week 52 in 2021, comprising 9100 detections in total), and the second data set, hereafter referred to as HPAI2122, contained data from 2021–2022 (week 39 in 2021 to week 49 in 2022, comprising 9376 detections in total). We included week 39–52 in 2021 in the HPAI1621 data set due to data constraints, thus the two datasets overlap in time. In the 2016–2021 data set, 33.6% of the detections were of the subtype H5N1 and 61.2% were of the subtype H5N8, whereas these proportions were 99.2% and 0.2% for the 2021–2022 data set (Supplementary Table S2).

### Endemic-epidemic time-series model

Testing multiple model formulations, we found that when comparing the baseline model with no seasonality (Baseline model 1 in Fig. 1) to models with one, two and three seasonal waves in both the endemic and epidemic (within-country effects) components, the best performing calibrated model for the HPAI1621 data (with the lowest scores for RPS and logS) was the model with three waves in the endemic component and one wave in the within-country effects in the epidemic component, and thus this model was chosen as the new baseline model (Base line model 2, Fig. 1, Supplementary Table S3). For the HPAI2122 data, 2 calibrated models had the exact same low logS score and included no seasonality in the endemic component and 2 and 3 waves in the within-country effects in the epidemic component respectively. We chose the simpler model with 2 waves in the within-country effects in the epidemic component as the new baseline model for HPAI2122 (Baseline model 2, Fig. 1, Supplementary Table S3). For HPAI1621, all models had calibration tests for RPS with *p* ≥ 0.05, indicating satisfactory calibration; however, for logS, not all models calibrated (*p* < 0.05, Supplementary Table S3). For the HPAI2122 data, only models with no endemic waves were calibrated (*p* ≥ 0.05), which was also the case for logS, except for one model including three waves in the endemic component and no waves in the epidemic component (*p* ≥ 0.05, Supplementary Table S3). Comparing the new baseline models (Baseline model 2 in Fig. 1) to models in which we added area of waterways/wetlands and length of coastline as log-transformed covariates or offsets, the calibrated model with the lowest scores for both RPS and logS was a model with covariates in the endemic component and no covariates or offsets in the epidemic components for the HPAI1621 data; this was chosen as the new baseline model (Baseline model 3 in Fig. 1). For the HPAI2122 data, the best performing calibrated model based on logS scores included no covariates or offsets in either component (Supplementary Table S3). This model was chosen as the new baseline model for HPAI2122 (Baseline model 3 in Fig. 1). Most of the HPAI1621 models calibrated based on RPS scores but not logS scores, particularly not models incorporating the covariates as offsets (*p* < 0.05, Supplementary Table S3). For HPAI2122 most of the models calibrated based on RPS scores, except for models with offsets in the endemic component. For logS, all models calibrated (*p* ≥ 0.05).

Adding spatial weights *w*_*ji*_ as a power-law model with distance-decay and models with country-specific correlated and uncorrelated random effects to the new baseline model (Baseline model 3 in Fig. 1) resulted in a best performing calibrated model that included the spatial weights power law and uncorrelated random effects for both the HPAI161 and HPAI2122 data sets (Supplementary Table S3). For both RPS and logS, most models for the HPAI1621 data calibrated (*p* ≥ 0.05), except for models without power law added to the spatial weights for logS (*p* < 0.05); the model with power law added, but no random effects; and the model with only correlated random effects and no power law added for RPS (*p* < 0.05). For the HPAI2122 dataset, only the models with no power law added and random elements did not calibrate for RPS scores (*p* < 0.05), whereas all models according to logS scores calibrated.

**Figure 3:**
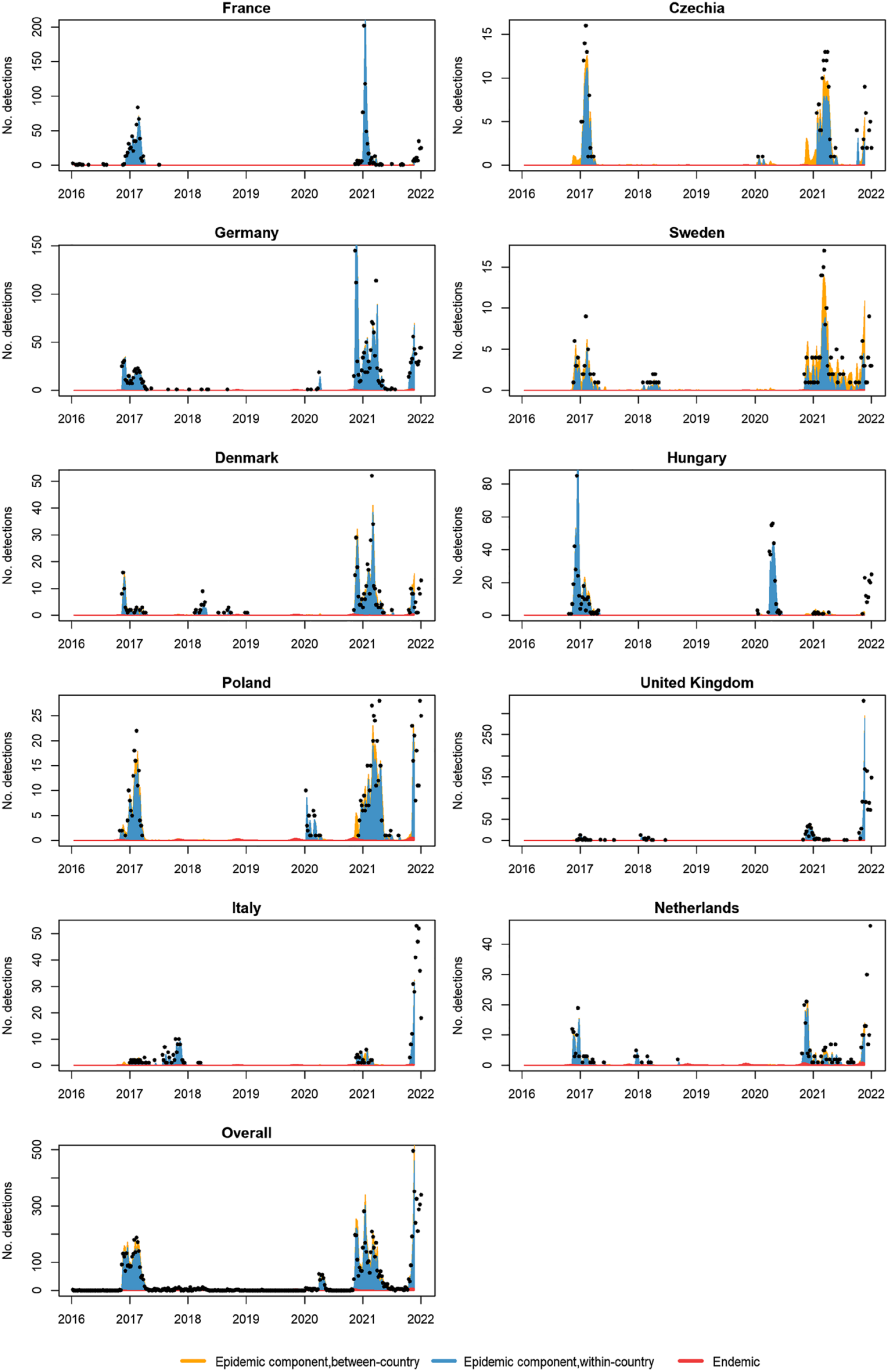
Country-wise model fit, and the relative contribution of model components based on the final multivariate time-series models for the HPAI1621 data. Dots show the actual counts of reported highly pathogenic avian influenza (H5 subtype) detections in domestic and wild birds. Only countries with > 200 detections are depicted. The last panel shows the overall model fit aggregated over all the 37 countries. Note that the scales on the Y-axes are different for some of the graphs, and zero/missing detections have been omitted. Although actual counts from week 47–52 in 2021 are depicted, they were not part of the training set in the model, and thus are not part of the model fit.

**Figure 4:**
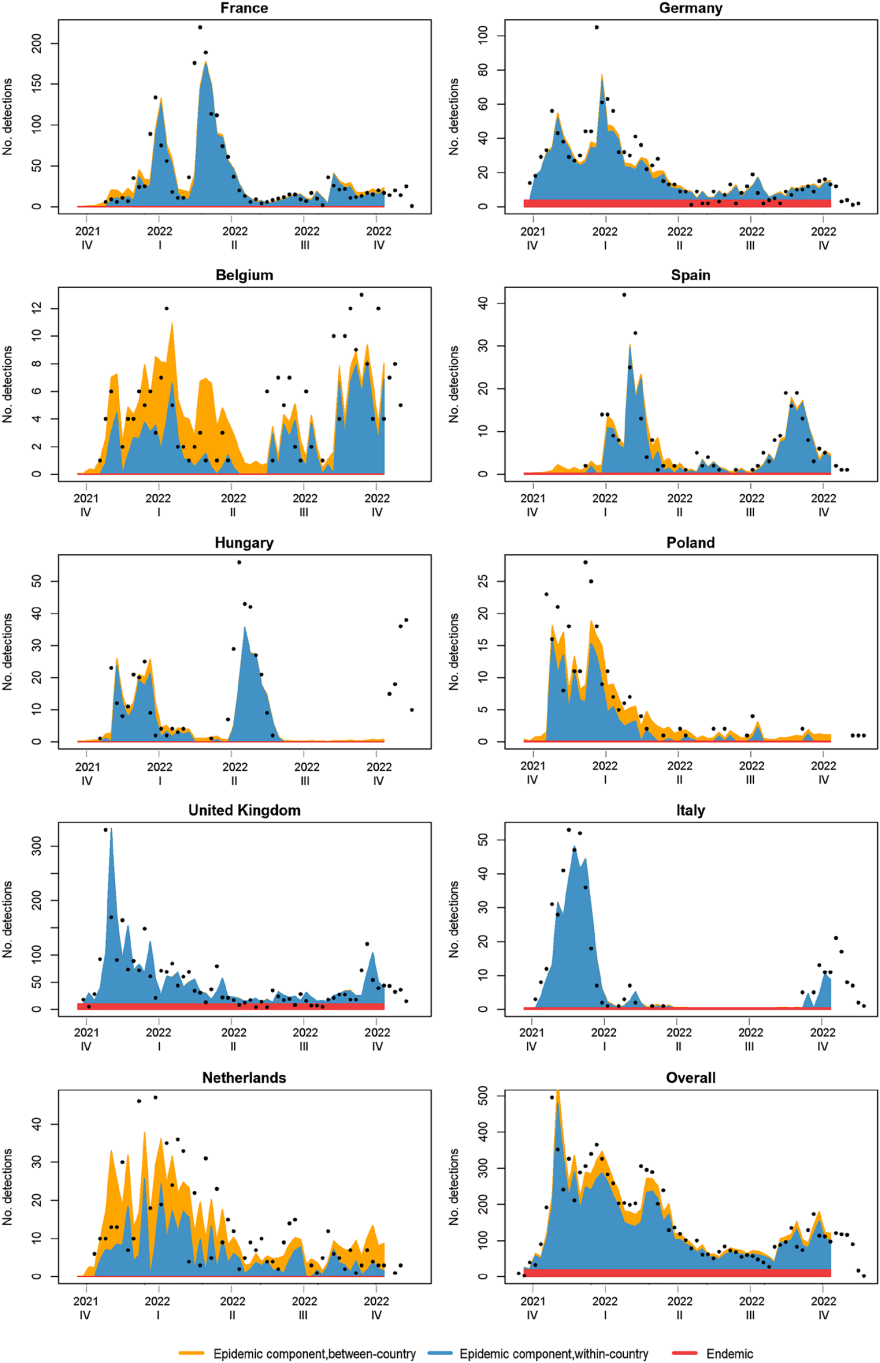
Country-wise model fit, and the relative contribution of model components based on the final multivariate time-series models for the HPAI2122 data. Dots show the actual counts of reported highly pathogenic avian influenza (H5 subtype) detections in domestic and wild birds. Only countries with > 200 detections are depicted. The last panel shows the overall model fit aggregated over all the 37 countries. Note that the scales on the Y-axes are different for some of the graphs, and zero/missing detections have been omitted. Although actual counts from week 44–49 in 2022 are depicted, they were not part of the training set in the model, and thus are not part of the model fit.

The final models for HPAI1621 and HPAI2122 both included power law and uncorrelated random effects. The HPAI1621 model included three seasonal waves in the endemic component and one wave in the within-country effect in the epidemic component as well as covariates in the endemic component, whereas the HPAI2122 model only included epidemic seasonality. The seasonality waves for both the endemic (3 waves) and epidemic component (2 waves) for the HPAI1621 model and the epidemic seasonality waves (2 waves) for the HPAI2122 model can be seen in the Supplementary Fig. S3. There are large peaks in AIV predictions during the autumn and smaller peaks during spring (Supplementary Fig. S3); but in the endemic component, there is a small peak in early summer for the endemic seasonality in the HPAI1621 model. For the within-country epidemic seasonality, we see large peaks in autumn and spring for the HPAI1621 model, but a large peak starting earlier during the summer season for the HPAI2122 model. We also see a very small, almost negligible, peak in the early spring. Epidemic between-country predicted transmission was mostly seen in Czechia, Sweden, and Poland in 2016–2021 (Fig. 3), compared to 2021–2022 where it was mostly in Poland, Belgium, and the Netherlands (Fig. 4).

The point estimates of the final HPAI1621 model show that length of coastline has a positive association with detections in the endemic component, whereas area of wetlands has a negative association (Table 2). However, the confidence intervals for these coefficients overlap zero (Table 2). Our final model for HPAI1621 suggests that most of the HPAI detections reported in Europe are epidemic (occasional localized outbreaks) in nature (94.6%), with 78.8% within-country and 15.8% between-country transmission; 5.4% of the reported detections were endemic (baseline rate comprising unreported outbreaks within a country and outbreaks originating from transmission from countries not included in our study) in nature. Compared to the HPAI1621 model, the model for HPAI2122 suggested that more HPAI detections were endemic in nature (12.2%), with 87.8% being epidemic in nature (73.3% within-country and 14.5% between-country transmission). The prediction plots show that there is a slight tendency for our final models to underestimate the number of detections in periods with many detections (Figs. 3 and 4).

**Table 2:** Coefficient estimates from the final multivariate time-series models for HPAI1621 and HPAI2122.

Model components/ parameters	HPAI1621				HPAI2122			
	Coeff	SE	2.5% CI	97.5% CI	Coeff	SE	2.5% CI	97.5% CI
*Endemic component*								
Sine(2*π*t/52)	0.646	0.089	0.471	0.821	NA	NA	NA	NA
Cosine(2*π*t/52)	2.099	0.266	1.577	2.621	NA	NA	NA	NA
Sine(4*π*t/52)	0.604	0.077	0.453	0.754	NA	NA	NA	NA
Cosine(4*π*t/52)	0.872	0.112	0.652	1.091	NA	NA	NA	NA
Sine(6*π*t/52)	1.398	0.174	1.058	1.738	NA	NA	NA	NA
Cosine(6*π*t/52)	0.689	0.096	0.522	0.856	NA	NA	NA	NA
Length of coastline	0.027	0.120	− 0.209	0.262	NA	NA	NA	NA
Area of wetland	− 0.168	0.214	− 0.588	0.252	NA	NA	NA	NA
Random intercept	0.669	1.016	− 1.323	2.660	5.163	1.890	1.458	8.867
*Epidemic component*								
Sine(2*π*t/52)	0.862	0.091	0.684	1.040	0.976	0.146	0.691	1.262
Cosine(2*π*t/52)	1.322	0.166	0.997	1.648	1.297	0.146	1.011	1.582
Sine(4*π*t/52)	NA	NA	NA	NA	0.954	0.106	0.746	1.162
Cosine(4*π*t/52)	NA	NA	NA	NA	1.029	0.104	0.825	1.233
Length of coastline	NA	NA	NA	NA	NA	NA	NA	NA
Area of wetland	NA	NA	NA	NA	NA	NA	NA	NA
Within-country random intercept	0.603	0.102	0.402	0.804	0.662	0.159	0.351	0.974
Between country random intercept	0.123	0.027	0.070	0.175	0.070	0.022	0.027	0.112
Spatial weights (*w*_*ji*_)	25.167	6.437	12.550	37.785	28.940	18.993	-8.286	66.165

HPAI1621 is the dataset containing detections from 2016 and 2021, whereas HPAI2122 is the dataset containing detections from 2021 and 2022. Both models include area of country relative to total area of all countries as offset in the endemic component, spatial weights *w*_*ji*_ as a power-law model with distance-decay for the between-country effects of the epidemic component and uncorrelated random intercepts for all components

*Coeff*. Coefficient estimate, *SE* Standard error.

In the HPAI1621 model, the exponentially transformed deviations of the random intercepts, indicating the model fit for each country, show that for the epidemic within-country random intercepts, particularly France, Switzerland, Hungary, and Germany (Supplementary Fig. S4A, pink colour) had more detections from within-country transmission than explained by the model. For the HPAI2122 model, countries with the largest random epidemic, within-country intercepts were France and Slovenia, followed by the UK, Italy, Hungary, and Spain (Supplementary Fig. S4B). Maps of the random intercepts for epidemic between-country transmission show that for the HPAI1621 model, countries such as the UK, Estonia, Sweden, and Bulgaria followed by Hungary received more cases from other countries than the model could explain (Supplementary Fig. S4A), whereas for the HPAI2122 model the highest random intercepts were found for the Netherlands, Hungary, France, and Poland (Supplementary Fig. S4B). As for the map of the endemic random intercepts, reflecting endemic circulation and transmission by migratory birds from countries not included in this study, countries in cyan in Supplementary Fig. S4A and B exhibit a relatively lower endemic incidence than the model predicted.

Fan plots show how well the final models performed for successive one-step-ahead predictions (one-week-ahead) using the test data sets (unseen data). We see that both models predict detections reasonably well, but there seems to be a lag of one week for some detection peaks (Supplementary Figs. S5 and S6). Comparing the final models to the original baseline model 1, shows how the overall predictions from the final HPAI1621 model encompass slightly more of the actual observed detections than the overall predictions from baseline model 1 (Supplementary Fig. S7). Furthermore, the HPAI2122 final model seems to predict more detections to be epidemic between-country and endemic in nature compared to Baseline model 1 (Supplementary Fig. S8).

**Figure 5:**
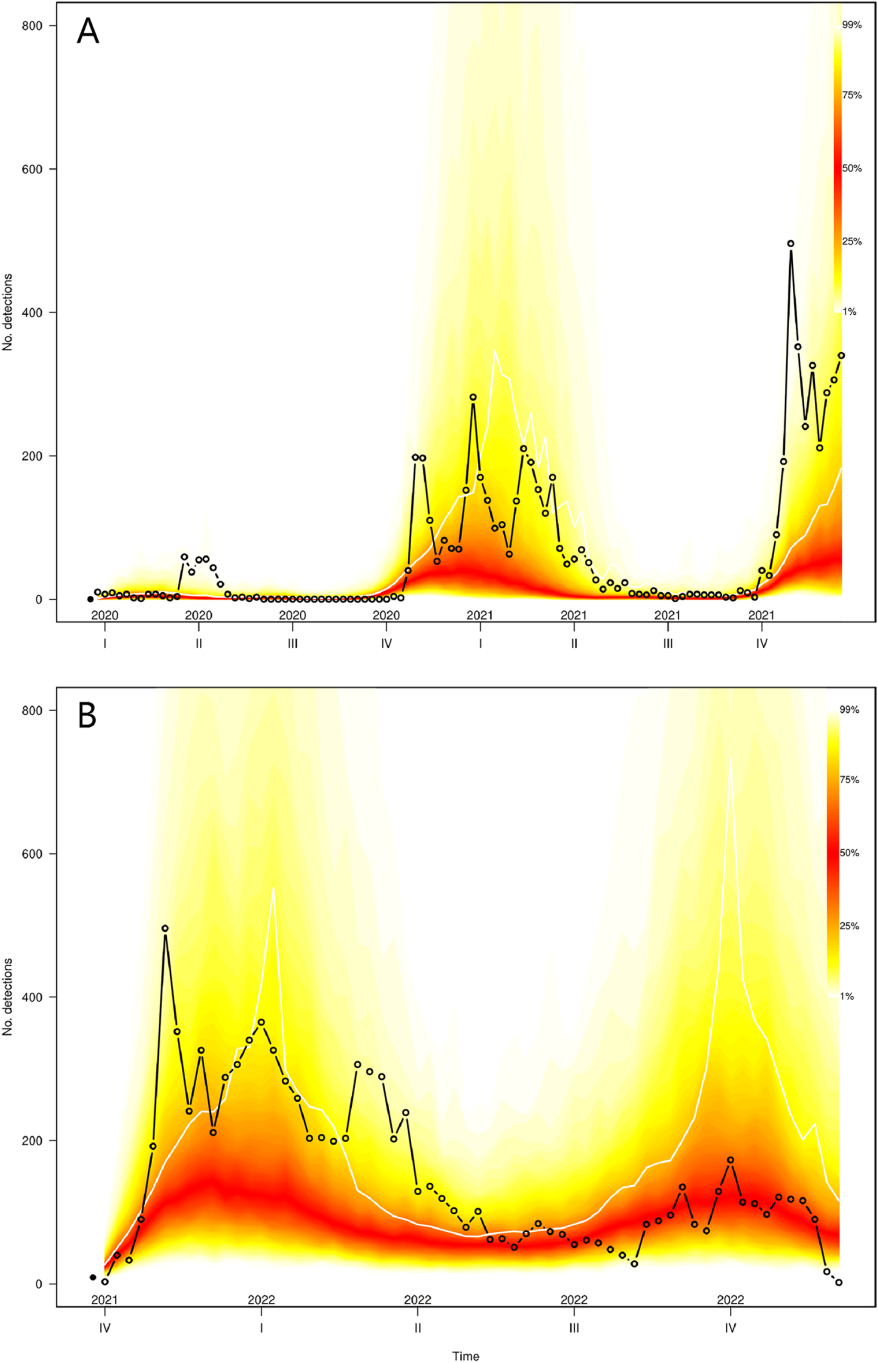
Simulation-based long-term forecast for (**A**) the HPAI1621 final model starting from the last week in 2019 (left-hand dot), and (**B**) the HPAI2122 final model starting from week 39 in 2021. The plots show weekly number of predicted and observed highly pathogenic avian influenza (H5 subtype) detections aggregated over all countries. The fan charts represent the 1% and 99% quantiles of the simulations (N = 500) each week; their mean is displayed as a white line. Actual reported number of detections are depicted with open circles. Data from week 47 to week 52 in 2021 for the HPAI1621 model and week 44–49 in 2022 in the HPAI2122 model were not used to train the models.

Using the final HPAI1621 model to make long-term forecasts from 2020 to 2021, Fig. 5A shows how the model predicted many detections in the period late 2020/early 2021 and late 2021 where a large number of detections were reported. However, it did not capture well the observed peak in detection in the spring 2020, following a period with low numbers of observed detections (early 2020). This indicates that the model forecasts relatively well in years with many detections, but less well in years following years with no or low observed detections. The mean size of all the simulated detections (N = 5925) was slightly smaller than the observed number of detections (N = 6593) from 2020–2021. The same pattern was seen for individual countries (Supplementary Fig. S9). For the HPAI2122 model, forecasting the years 2021–2022 resulted in continuous predicted detections throughout the period, which corresponds to observed number of predictions. The model showed two peaks in late 2021/early 2022 and again in late 2022, and whereas the model predicted the increase in 2021/2022 detections fairly well, it overshot predictions in late 2022 (Fig. 5B). For individual countries we see the same patterns (Supplementary Fig. S10). For the HPAI2122 model, the mean size of simulated detections (N = 11,710) was larger than the actual number of detections during 2021–2022 (N = 9367).

Aggregating the data from 2016 to 2021 bi-weekly and following the same model selection process as above resulted in a final model very similar to the original weekly aggregated HPAI1621 model, however with 2 seasonal waves in the endemic component and three seasonal waves in the epidemic component, covariates in both the endemic and epidemic components, spatial weights power law and uncorrelated random effects (Supplementary Tables S4 and S4 and Fig. S11).

## Discussion

We here utilized a time-series modelling framework to predict country level detection of H5 HPAI based on freely available WOAH-WAHIS HPAI data. This enables the greatest use of data from other, surrounding countries for continuous risk assessment, taking endemic and epidemic transmission into account. Furthermore, we identified a difference between the model fitted to 2016–2021 data and the model fitted to 2021–2022 data: in the latter the seasonal component was weaker, reflecting a concerning change towards reduced seasonality in occurrence of HPAI.

Overall model fits showed a fair congruence with the reported data for all countries over time (Figs. 3 and 4). The final models encompassed more of the observed detections compared to the original baseline models, particularly for the HPAI1621 model, so that adding covariates (for HPAI1621 only), long-range transmission and random effects improved the model fit. Even though the endemic detections became more frequent in the model fitted to 2021–2022 data compared to the model fitted to 2016–2021 data, the final models generally predicted fewer endemic than epidemic detections; this could be due to more detections being explained by the epidemic long-distance transmission and/or epidemic random effects. The final model fits showed that the model framework is sufficiently flexible to predict detections in countries with large temporal variation in the number of HPAI detections (Figs. 3 and 4). When used for predicting the number of detections in the following week, the models were reactive to the reported data from each country; they can miss the start of periods with many sudden detections, such as in France and Hungary, but then the probability estimates were adjusted to the new level based on the previous week´s detection data, which is a common property of one-step ahead forecasting^33^ (Supplementary Figs. S5 and S6). During prolonged periods with detections, the models appeared to predict the overall risk well, even when there was a declining trend (e.g., Germany and Denmark, Figs. 3 and 4). Further studies with more, diverse data are needed to investigate these properties of the model.

A change in the seasonality of HPAI in Europe has been suspected as detections have been observed during the summer months in 2021 and particularly in 2022, in contrast to previous years^12,13^. Creating separate models for 2016–2021 and 2021–2022 produced models with different seasonality, particularly in the endemic component. According to the HPAI1621 final model, the 2016–2021 detection data were best described by a model with three seasonal waves in the endemic component and one seasonal wave in the epidemic component (HPAI1621 model). The model for 2021–2022 (HPAI2122) included no seasonality in the endemic component (and no covariates), instead assuming a more or less constant endemic contribution to predicted detections. The HPAI2122 model furthermore included two seasonal waves in the within-country effects of the epidemic component. The final HPAI1621 model generally predicted a higher probability of detections during the winter months but did not predict well for early 2020 following a period with a low number of recorded detections. However, in years in which detections were common (e.g., winter 2020/2021 and last quarter of 2021), the model captured the increased magnitude of detections. The same outcome was found for individual countries. The model incorporates a strong seasonality effect, captured by the seasonal waves in the final model. The epidemic seasonal wave likely reflects HPAI transmission from migratory birds from countries within our study, and thus the one wave in the final model may reflect the timing of the main bird migration periods within countries (winter/spring). The endemic component, represented by three waves in the final model, might indicate that more bird species or migration events are involved in long-distance transmission (from countries not included in this study), and likely captures transmission related to wild bird behaviour (e.g., contact during mating season or communal roosts during winter) combined with the seasonal survival rate of the virus in the environment. Further studies should investigate if these wave patterns differ between countries, reflecting differences in migratory bird species, local bird behaviour or potentially farm-to-farm transmission. The seasonal component in the model reflects the general conditions for HPAI to occur, which between 2016 and 2021 were strongly seasonal in Europe. The final HPAI2122 model predicted continuous detections throughout late 2021 and all of 2022. The model generally predicted well for the 2021/2022 winter season and throughout the summer but predicted more detections than observed in the autumn of 2022. The endemic component included no seasonality, and thus the model assumed a constant contribution from the endemic component. Thus, any seasonality seen in the model predictions were due to the two seasonal waves in the within-country effects of the epidemic component. As with the HPAI1621 model, this epidemic seasonality could be due to HPAI transmission from migratory birds from countries within our study or due to general bird behaviour. The lack of seasonality of the endemic component could also be due to this component not solely being related to migratory events, indicating that detections were observed throughout the year. Countries such as France, Germany, UK, and Denmark have had outbreaks of HPAI in domestic poultry and ducks during the summer months^53^. Some of these outbreaks, particularly in France and Hungary, were caused by between-farm spread and thus not related to migratory birds^53^. Thus, these models, and perhaps particularly the HPAI2122 model, are useful for assessing the general risk and timing of HPAI occurrence within individual countries on a weekly basis. However, it should be kept in mind that the occurrence of HPAI also relies on stochastic events such as bird migration timing, which is heavily influenced by the weather^54^. Seeing as how the seasonality of HPAI detections have changed since 2016, it is important to continuously update the models to reflect the current situation and seasonality. Extrapolation of this modelling framework to other continents is an area of research that could be pursued if appropriate surveillance data are available.

Geographically, the models both over- and underestimated HPAI detections within countries. Underestimation (RI > 1) was most pronounced for Germany, Switzerland, Hungary, and France for the HPAI1621 model and for France, the UK, Spain, Italy, Slovenia, and Hungary for the HPAI2122 model, in which more detections attributed to within-country transmission were observed (Supplementary Fig. S4). Overestimation (RI < 1) of detections attributed to within-country transmission mostly occurred in countries located at the edge of the study area, e.g., Norway, Finland, and Ukraine for both the HPAI1621 and HPAI2122 models. This could indicate that for countries with missing information on detections in some of their neighbouring countries, the epidemic within-country estimates capture more detections than for other countries with neighbour data i.e., an edge effect. This also means that the model will not incorporate, nor predict, the magnitude of outbreaks where more birds have been affected than those reported to WOAH-WAHIS. The HPAI1621 model underestimated (R < 1) the between-country transmission in countries such as UK, Sweden, Finland, Estonia, Hungary, and Bulgaria whereas for the HPAI2122 model, this was especially seen for the Netherlands, Hungary, France, and Poland. Most of these countries have a considerable length of coastline, which might attract migratory birds from areas other than the directly neighbouring countries (but still only the countries included in this study). This could possibly create noise in the estimated between-country effect. The endemic component here reflects all transmission not explained by within- and between-country spread, e.g., transmission from unreported outbreaks within each country or from migratory birds from countries not included in this study. For a large number of countries, the endemic component underestimated (R > 1) the number of detections, particularly for the HPAI2122 model (Supplementary Fig. S4). This could be due to underreporting, or reflects that for these countries, the model attributes more detections to surrounding countries (for which this information exists in the data), reducing the estimated effect of migratory bird transmission. Many of the countries where the endemic component underestimated the number of detections lie on several bird migratory flyways with many different migrating birds of different origins^55–57^. Adding data on bird migration routes as covariates in our model could potentially improve the predictive power, but such data might be difficult to obtain and manage.

We found that including the covariates length of coastline and area of waterways/wetlands improved the HPAI1621 model, indicating an association between detection of HPAI virus and these covariates. However, the point estimates for both these covariates overlap zero, so the effect is statistically non-significant. Several other studies have found an effect of landscape on AIV occurrence in wild and domestic birds. In a previous study in Denmark, using mixed model generalized linear models, Kjær et al.^6^ found an association between distance to coast and distance to wetlands and AIV in wild birds collected through passive surveillance. In Romania, Ward et al.^58,59^ found a relationship between HPAI outbreaks and distance to migratory waterfowl sites, distance to major roads and distance to rivers or streams using conditional logistic regression models and spatial lag regression models. Using a machine learning (ML) approach, Belkhiria et al.^42^ found that land cover and distance to coast were important when predicting risk areas for AIV in wild birds in California. On a large scale, Walsh et al.^60^ used data on HPAI occurrence reported to the WOAH (as we did in the present study) in addition to spatial surface water data and domestic bird density data. They analysed the data using machine learning algorithms and found a spatio-temporal pattern of HPAI occurrence in domestic poultry throughout the world. On a smaller scale, Schreuder et al.^61^ used a machine learning algorithm and data on wild bird densities and landscape variables to spatially predict HPAI in the Netherlands with high accuracy. Pereira et al.^62^ used a fireworks-like surveillance approach to fit a model to WOAH-WAHIS data for HPAI H5N1. This model was able to describe the data well but did not include a component to account for detections not reported to the WOAH within or outside the study area.

The model framework used here has some limitations for the purpose of modelling AIV. Firstly, the data do not account for differences in detection effort, sampling scheme or prevention measures in the different countries reporting detections. Incorporating such information into this modelling framework could potentially improve our models, although it can be complicated to compare and categorize surveillance systems between countries and time periods. Secondly by combining HPAI detections in both domestic and wild birds, the models assume that HPAI can spread from wild to domestic birds and vice versa. This may not be the case, mainly due to farm biosecurity practices but also due to infected domestic birds usually being culled and thus not able to transmit HPAI to their surroundings. We did first attempt to run separate models for domestic and wild birds, but data constraints caused the models to not to calibrate. The seasonal detection patterns in Fig. S2 (Supplementary Material) suggest that combining the data is warranted, as domestic and wild birds exhibit similar seasonal patterns. Thus, we hypothesize that the general transmission in an area is in most cases high when there is a spill over to domestic birds, and an infected poultry flock reflects transmission in the wild bird population, which can transmit to areas in the same country as well as other countries. Also, when included in the model components, the models assume a constant contribution from seasonality every year; for example, there is a probability of detection in winter even in years without any detections. This seasonality effect might change over time as we have seen in the HPAI2122 model, for instance due to climate change (as climate has an impact on bird migration and the timing thereof), and the circulating HPAI strains (likely with different properties and target species) can change over time; therefore, model fit might be reduced if it is fitted to a long period of data. Ideally, a model should be able to incorporate a trend over time so that seasonality changes and other changes related to climate can be captured. Furthermore, we did not differentiate between different H5 HPAI viruses, due to data constraints. However, different AIV strains may have different transmission potentials^63^, affect different species and thus have different transmission patterns. The 2016–2021 detection data consisted mainly of H5N8, whereas the 2021–2022 detection data consisted mainly of H5N1. In addition, more than 20 genotypes have been detected in Europe since 2016, which could explain some of the differences between the models. If more data becomes available in the future, it could be feasible to apply our models on specific subtypes and genotypes separately. This could potentially improve the predictive ability and the separation into the endemic and epidemic components of the modelling framework. Furthermore, detections of one strain in an area are logically not directly related to other strains in other areas (as in having been transmitted between the areas). However, within each season, detections in the countries included in this study have up until December 2022 been dominated by the same few subtypes in all countries. Another limitation is that the models cannot account for differences in reporting effort, which likely differs between countries and changes over time. However, this remains a challenge for all model types, and cannot easily be separated from the general level of occurrence within each country. Both models estimated that the vast majority of detections were epidemic in nature and could be explained by either within-country or between-country transmission. This shows that the models explained the majority of detections based on previous within-country transmission, with a contribution from between-country transmission. However, whereas only 5.4% of the detections were attributed to endemic (unexplained) transmission in the HPAI1621 model, this percentage was 12.2% in the HPAI2122 model. This suggests that the HPAI2122 model estimates rely more on unreported endemic HPAI virus circulating within the countries, and transmission from migratory birds coming from countries not included in our study. An explanation for this could be that the increased outbreaks during summer make it difficult for people to see the dead birds, due to foliage e.g., in forests. It could also reflect increased decomposition of carcasses at warmer temperatures, or increased activity in scavenger species, quickly removing carcasses and thereby reducing the detection. It could furthermore be that increasing numbers of outbreaks makes passive surveillance less sensitive as people may stop reporting when finding dead birds is no longer unusual. Furthermore, the model selection could have an impact on the resulting final model. We here chose to test seasonality as a starting point, but model selection could be sensitive to the order of variables included. This should be explored in future studies. Lastly, selection of training and test data for each of the data sets could impact model results, however, with these kinds of models it is a trade-off between having enough data to calibrate the models and using up-to-date data that captures recent changes in the detection patterns. The epidemiology of avian influenza has always been dynamic, and during the past few years we have seen an even more dynamic system than expected. Predicting such a dynamic system is challenging, and what we present here is one approach to this challenge.

Despite the challenges highlighted, these models represent a practical approach to modelling the risk of AIV occurrence, since there are many potential variables in the system for which we do not have information. Despite such challenges, this model finds use for informing policy makers about the HPAI spatiotemporal risk, using readily available surveillance data. In future research, more layers could be added to the model including the actual bird density, species composition and temperature related to bird behaviour and thus transmission.

## Conclusion

The model framework was able to use surveillance data on HPAI detections in European countries and could be useful for predicting the probability and timing of detections. Separating our data into the years 2016–2021 and 2021–2022 revealed a seasonal shift in observed and predicted detections in Europe. HPAI detections were mostly explained by within-country transmission, but with a considerable contribution from other countries. The model based on 2021–2022 data attributed more detections to endemic transmission than the model based on 2016–2021 data. A strong seasonal component reflected the large temporal variation. This modelling framework can be used as a decision support tool to predict periods with higher risk of HPAI within a country.

### Supplementary Information

Supplementary Information.

## Data Availability

Data retrieved from the World Organisation for Animal Health (WOAH) (2022) – *Periodical Data Extraction WAHIS SharePoint*. Retrieved on 2022–12-15 from https://oieoffice365.sharepoint.com/:x:/r/sites/PeriodicaldataextractionsOIE-WAHIS/Shared%20Documents/infur_20221209.xlsx?d=wbde66024d7754376b0dbf8080c12b41b&csf=1&web=1&e=23QwTb. Reproduced with permission. WOAH bears no responsibility for the integrity or accuracy of the data contained herein, but not limited to, any deletion, manipulation, or reformatting of data that may have occurred beyond its control. Data and model code of this study are available on figshare: https://doi.org/10.6084/m9.figshare.21975488.
