# Author Correction: Using surveillance data for early warning modelling of highly pathogenic avian influenza in Europe reveals a seasonal shift in transmission, 2016–2022

**DOI:** 10.1038/s41598-023-43740-4

**Published:** 2023-10-03

**Authors:** Lene Jung Kjær, Michael P. Ward, Anette Ella Boklund, Lars Erik Larsen, Charlotte Kristiane Hjulsager, Carsten Thure Kirkeby

**Affiliations:** 1 Department of Veterinary and Animal Sciences, Faculty of Health and Medical Sciences, University of Copenhagen, Copenhagen, Denmark; 2 Faculty of Science, Sydney School of Veterinary Science, University of Sydney, Camden, NSW Australia; 3 Statens Serum Institut, Copenhagen, Denmark

Correction to: *Scientific Reports* 10.1038/s41598-023-42660-7, published online 16 September 2023

The Acknowledgments section in the original version of this Article was omitted. The Acknowledgments section now reads:

“This work was carried out as part of the ENIGMA project, a veterinary contingency project funded by the Danish Veterinary and Food Administration.”

The original Article has been corrected.

