# Supplementary material for: Using surveillance data for early warning modelling of highly pathogenic avian influenza in Europe reveals a seasonal shift in transmission, 2016–2022
Source: Sci Rep. 2023 Sep 16;13:15396. doi: 10.1038/s41598-023-42660-7 (PMC10505205; doi:10.1038/s41598-023-42660-7)
Supplement: Supplementary file 1 — Supplementary Information. [file 41598_2023_42660_MOESM1_ESM.docx]

**Supplementary Information for**

**Using surveillance data for early warning modelling of highly pathogenic avian influenza in Europe reveals a seasonal shift in transmission, 2016-2022**

Lene Jung Kjær^1*^, Michael P. Ward^2^, Anette Ella Boklund^1^, Lars Erik Larsen^1^, Charlotte Kristiane Hjulsager^3^, and Carsten Thure Kirkeby^1^

^1^Department of Veterinary and Animal Sciences, Faculty of Health and Medical Sciences, University of Copenhagen, Denmark

^2^Faculty of Science, Sydney School of Veterinary Science, University of Sydney, Camden NSW, Australia

^3^Statens Serum Institut, Copenhagen, Denmark

*Corresponding author:

**Email:** [lenju@sund.ku.dk](mailto:lenju@sund.ku.dk)

**Adjacency matrix**

We obtained shapefiles of the European countries included in this study from Natural Earth^1^ and calculated an adjacency matrix to be used in a spatial weights matrix in the endemic-epidemic time-series model. This adjacency matrix calculated values for pairs of countries dependent on whether they were directly adjacent or whether they were connected through other countries; for example, Denmark and Germany share a border and would get a value of 1, whereas Portugal is connected to France through Spain, so here the value would be 2. Many countries in Europe are divided by waterways, which would automatically give the value zero in the adjacency matrix (for example Denmark and Sweden, and furthermore Iceland and Faroe Islands would stand alone with no adjacencies). Many bird species can easily traverse water separating country borders, and a lot of migrating water birds fly over large distances (for example between Iceland and Faroe Island to Great Britain and mainland Europe^2^). To account for this in the adjacency matrix, we modified the shape file of Europe to connect countries by adding water as a shape between these countries (Fig. S1). By doing this we connected Denmark to Great Britain, Norway, and Sweden. We also connected Sweden to Germany, the Baltic countries, and Poland, Finland to Estonia and Latvia, and Iceland and Faroe Island to Great Britain, Norway, and Denmark. We furthermore connected Great Britain to mainland Europe and connected Italy to the Balkans. Thus, these specified waterways will in the adjacency matrix act as a connecting country. We based these connections on migratory routes of many of the different migrating swan and goose species in Europe such as whooper swan (*Cygnus cygnus*), mute swan (*Cygnus olor*), tundra swan (*Cygnus columbianus*), barnacle goose (*Branta leucopsis*), Brent goose (*Branta Bernicla*), and Canada goose (*Branta canadensis*)^2^.


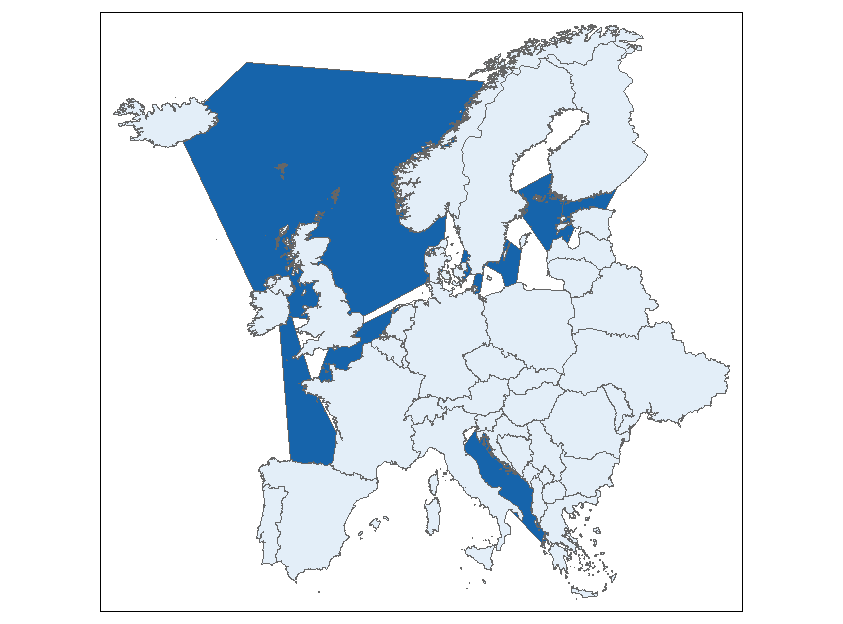


**Figure S1.** Waterway connections (dark blue) created for the spatial adjacency order of the European countries included in the endemic-epidemic time-series model in this study. The map was created using the package tmap^3^ in R 4.1.2^4^.


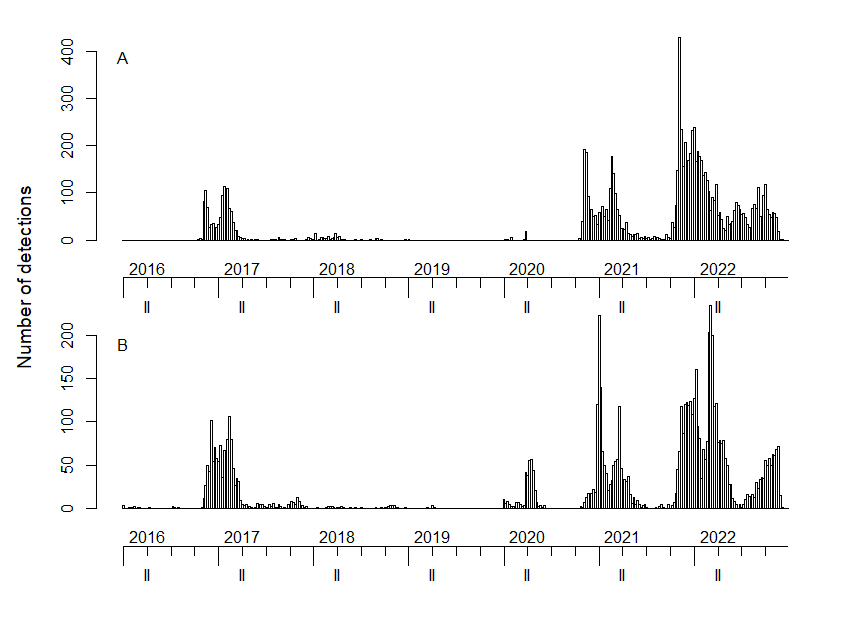


**Figure S2.** The number of reported highly pathogenic avian influenza (H5 subtype) detections in A) wild birds (N=8,870) and B) domestic birds (N=6,679) summed over 37 European countries (including Faroe Islands) and shown over time, 2016-2022.


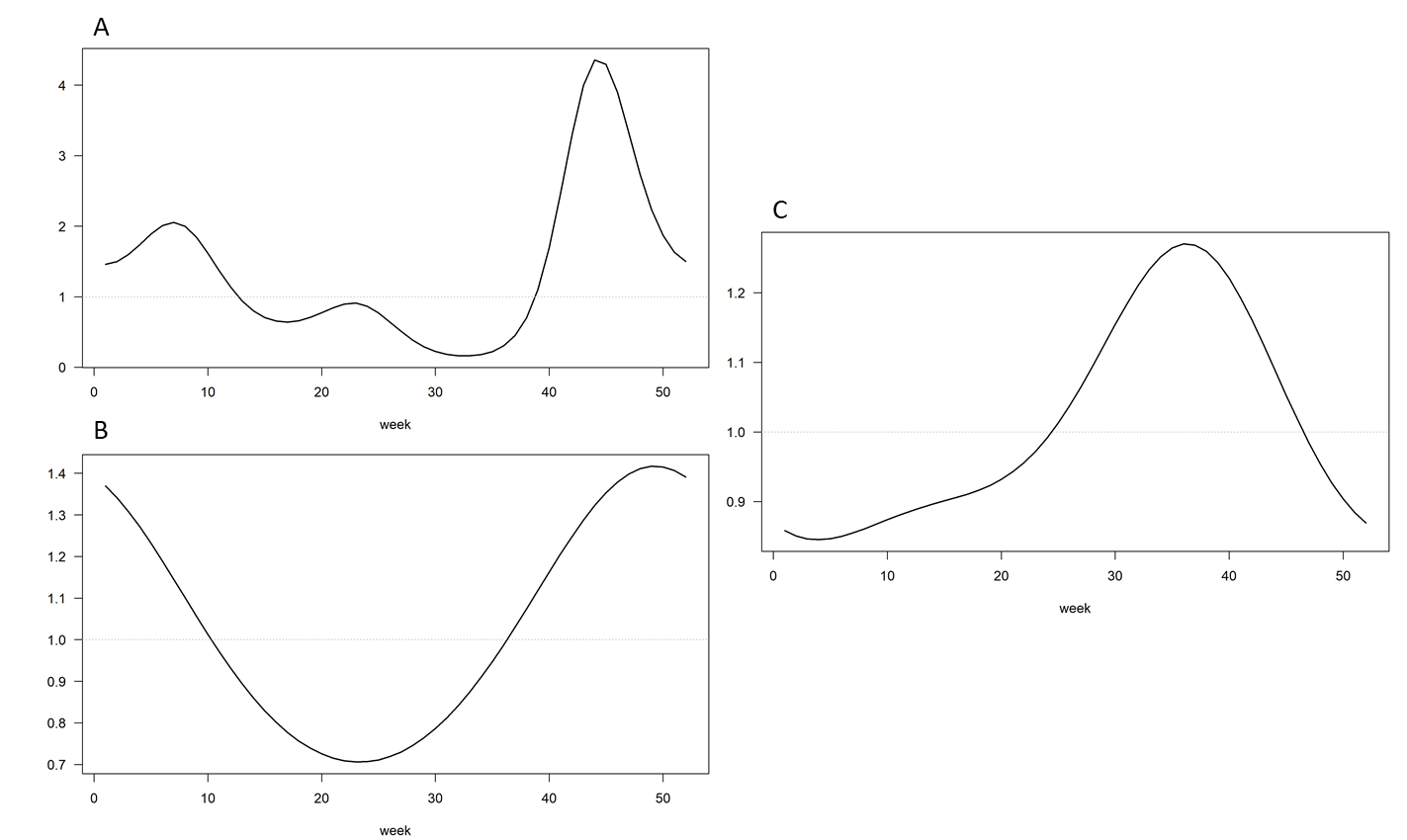


**Figure S3.** Seasonal waves (frequency ω = 2π/52) included in the final multivariate time-series models for A) the endemic component (3 waves) in the HPAI1621 model, B) the within-country effects in the epidemic component (2 waves) in the HPAI1621 model, and C) the within-country effects in the epidemic component (2 waves) in the HPAI2122 model.


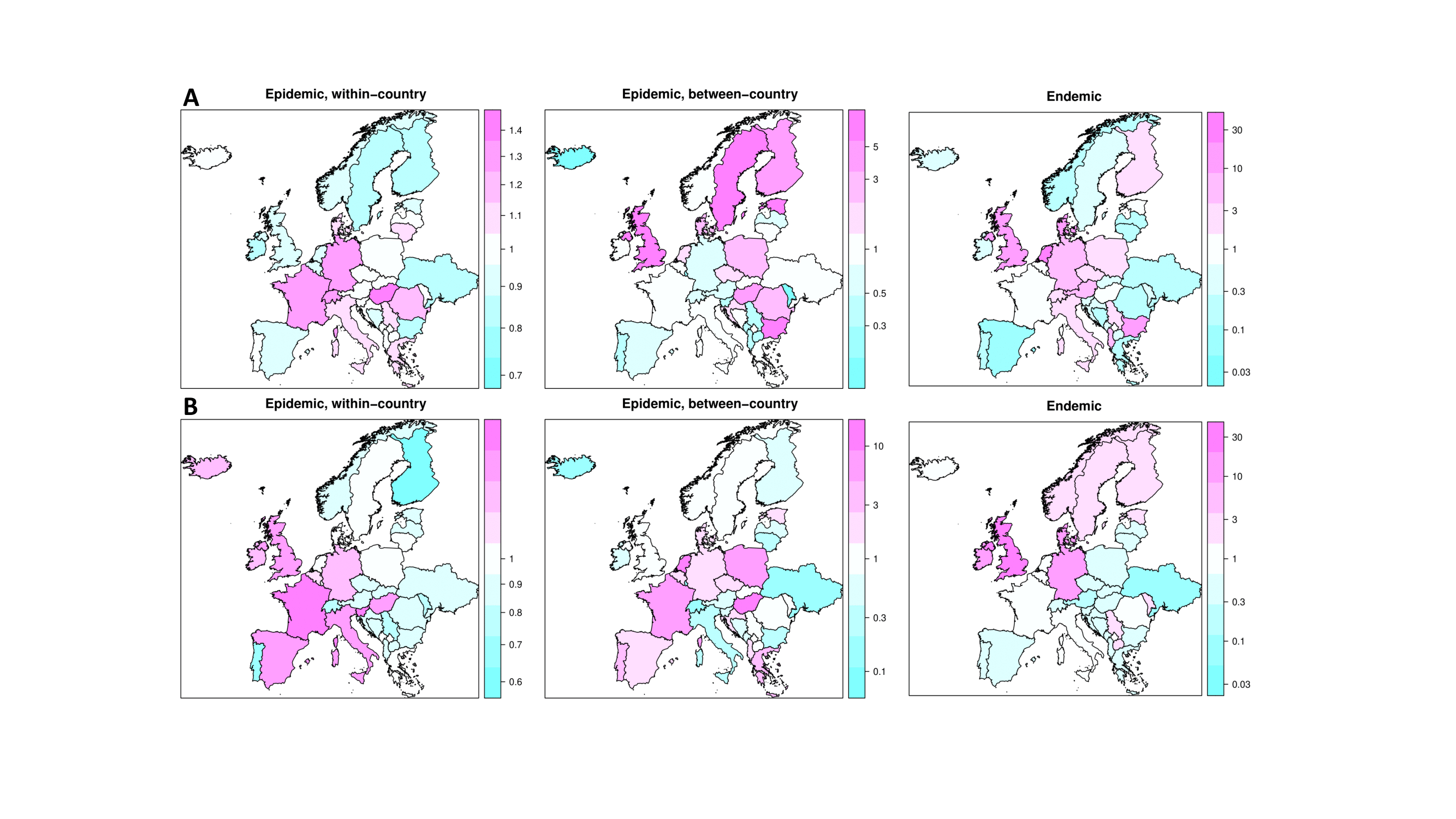


**Figure S4.** Epidemic (between and within country) and endemic random intercepts (RI) from A) the final HPAI1621 multivariate time-series model and B) the final HPAI2122 multivariate time-series model for all 37 countries. RI < 1 suggest lower number of detections than predicted. The maps were created using the package surveillance^5,6^ in R 4.1.2^4^.

**
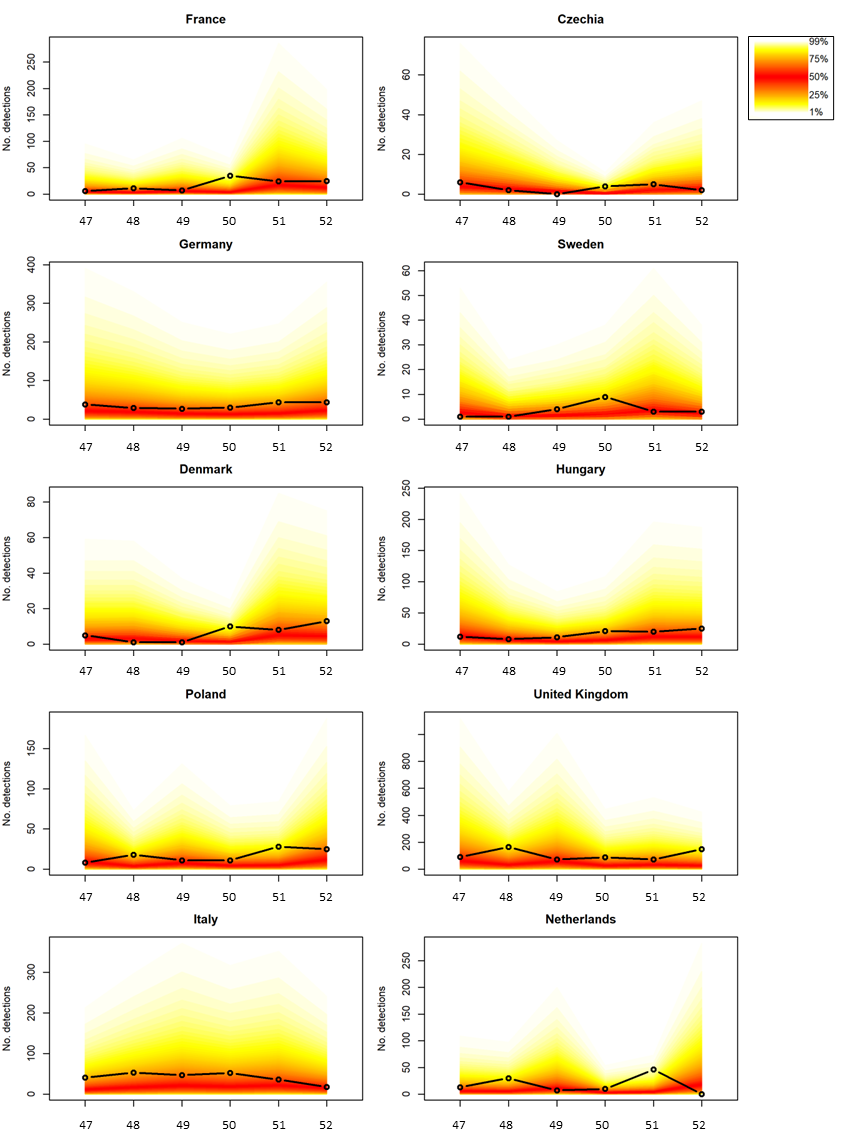
**

**Figure S5.** Fan plots of rolling one-week-ahead forecasts during week 47 to week 52 in 2021, produced by the final multivariate HPAI1621 time-series model. The X-axis shows the week number, and the Y-axis show the number of detections. Only countries with > 200 total highly pathogenic avian influenza (H5 subtype) detections are depicted. The fan chart represents the 1% and 99% quantiles of the simulations each week. Actual reported number of detections are depicted with open circles. Note that the scales on the Y-axes are different for some of the graphs.

**
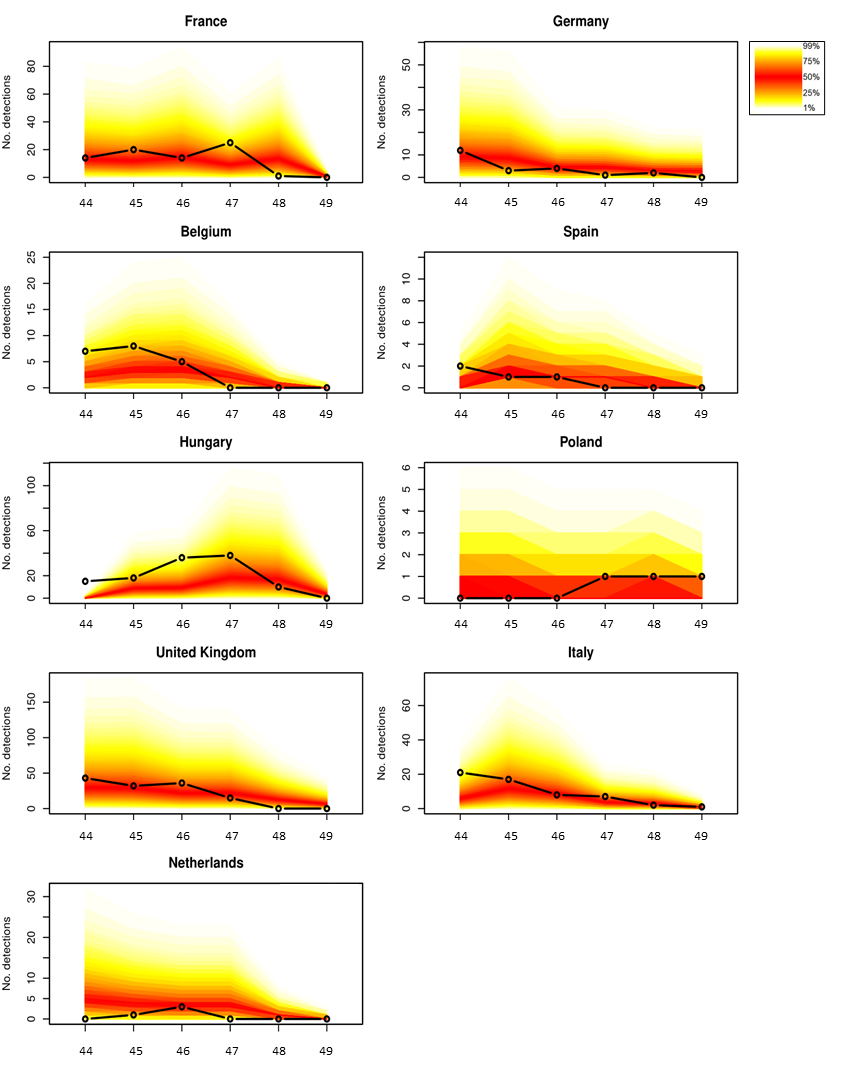
**

**Figure S6.** Fan plots of rolling one-week-ahead forecasts during week 44 to week 49 in 2022, produced by the final multivariate HPAI2122 time-series model. The X-axis shows the week number, and the Y-axis show the number of detections. Only countries with > 200 total highly pathogenic avian influenza (H5 subtype) detections are depicted. The fan chart represents the 1% and 99% quantiles of the simulations each week. Actual reported number of detections are depicted with open circles. Note that the scales on the Y-axes are different for some of the graphs.

**
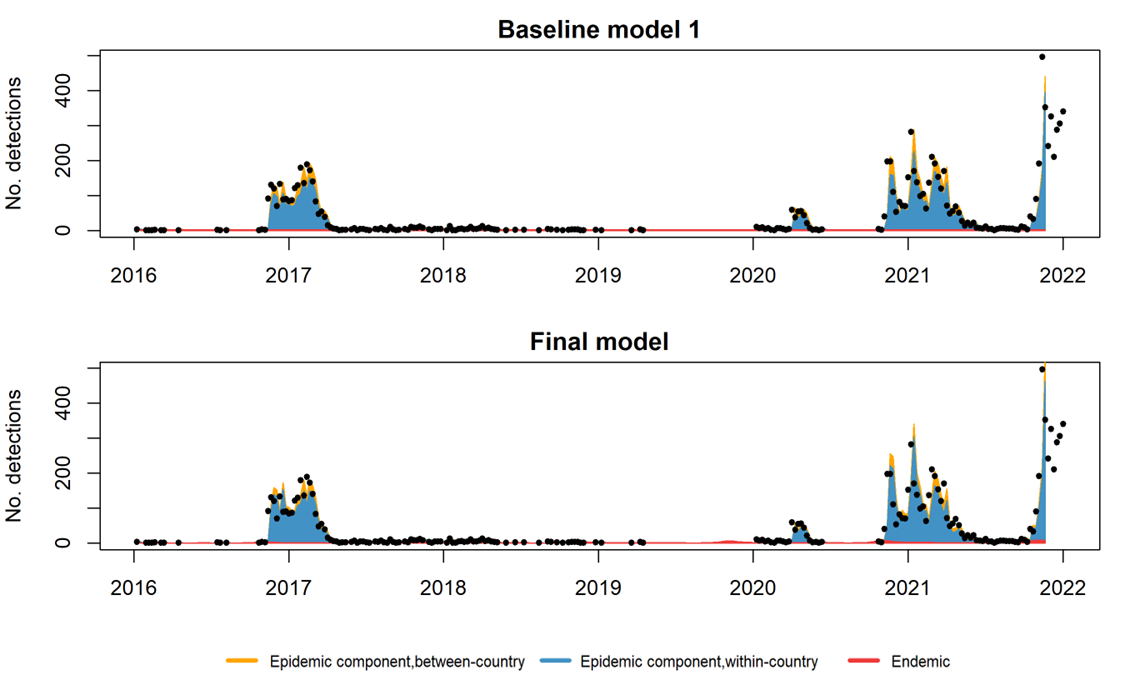
**

**Figure S7.** Overall model fit aggregated over all the 37 countries showing the relative contribution of model components for the HPAI1621 baseline model 1 and the HPAI1621 final multivariate time-series model. Dots show the actual counts of reported highly pathogenic avian influenza (H5 subtype) detections in domestic and wild birds. Although actual counts from week 47-52 in 2021 are depicted, they were not part of the training set in the model, and thus are not part of the model fit.

**
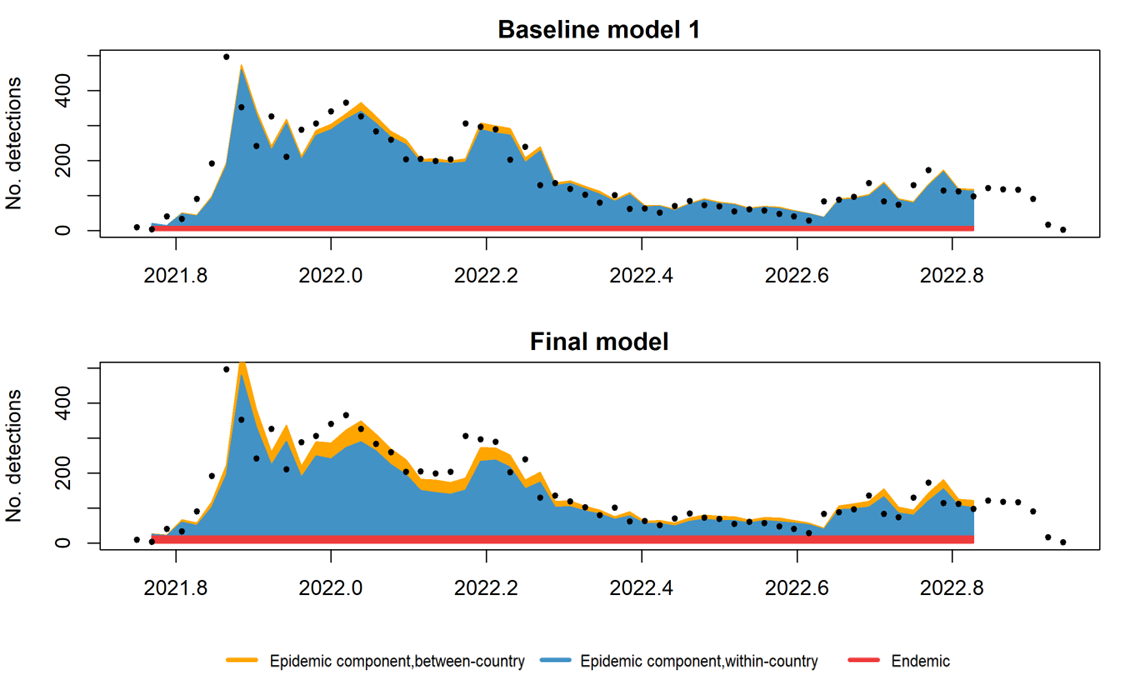
**
**Figure S8.** Overall model fit aggregated over all the 37 countries showing the relative contribution of model components for the HPAI2122 baseline model 1 and the final multivariate time-series model. Dots show the actual counts of reported highly pathogenic avian influenza (H5 subtype) detections in domestic and wild birds. Although actual counts from week 44-49 in 2022 are depicted, they were not part of the training set in the model, and thus are not part of the model fit.

**
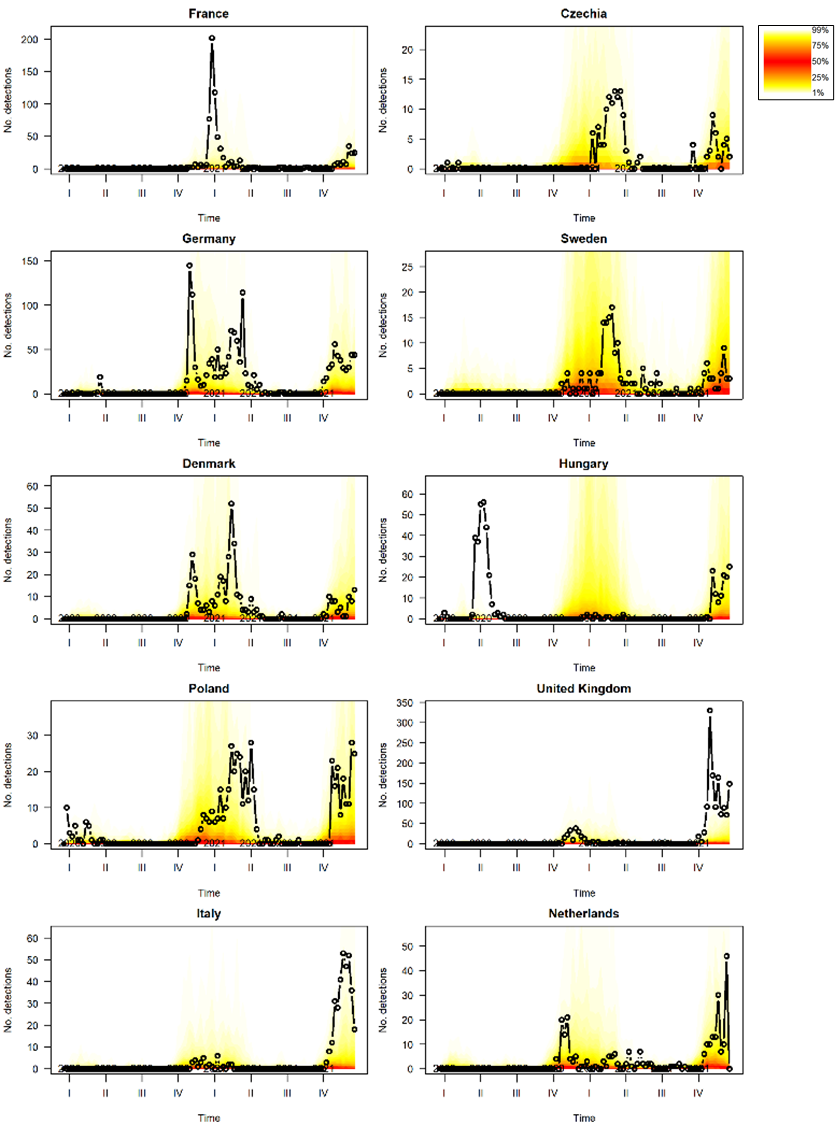
**

**Figure S9.** Simulation-based long-term forecast starting from the last week in 2019 (left-hand dot) to week 19 in 2022 using the HPAI1621 model. The plot shows weekly number of HPAI detections for countries with > 200 reported detections. The fan chart represents the 1% and 99% quantiles of the simulations each week. Actual reported number of detections are depicted with open circles. Data from week 47 to week 52 in 2021 were not used to train the model. Note that the scales on the Y-axes are different for some of the graphs.


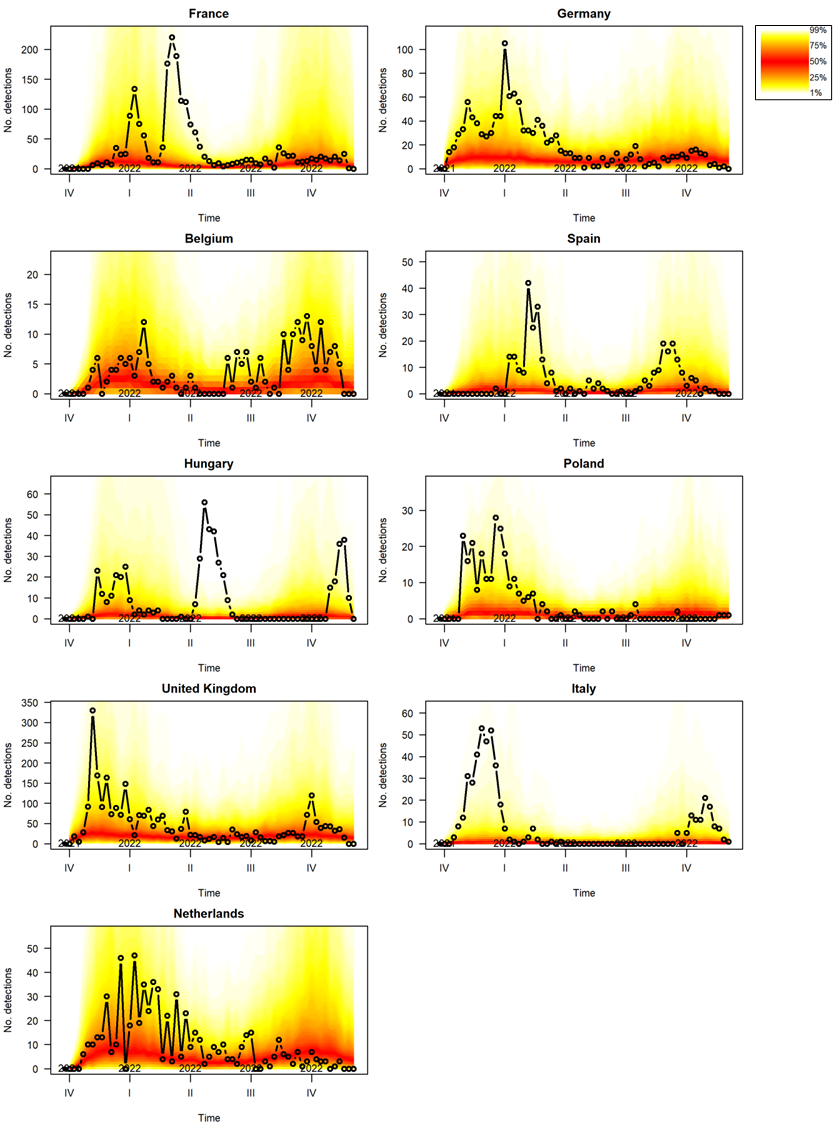


**Figure S10.** Simulation-based long-term forecast starting from week 39 in 2021 (left-hand dot) to week 49 in 2022 using the HPAI2122 model. The plot shows weekly number of HPAI detections for countries with > 200 reported detections. The fan chart represents the 1% and 99% quantiles of the simulations each week. Actual reported number of detections are depicted with open circles. Data from week 44 to week 49 in 2022 were not used to train the model. Note that the scales on the Y-axes are different for some of the graphs.

**
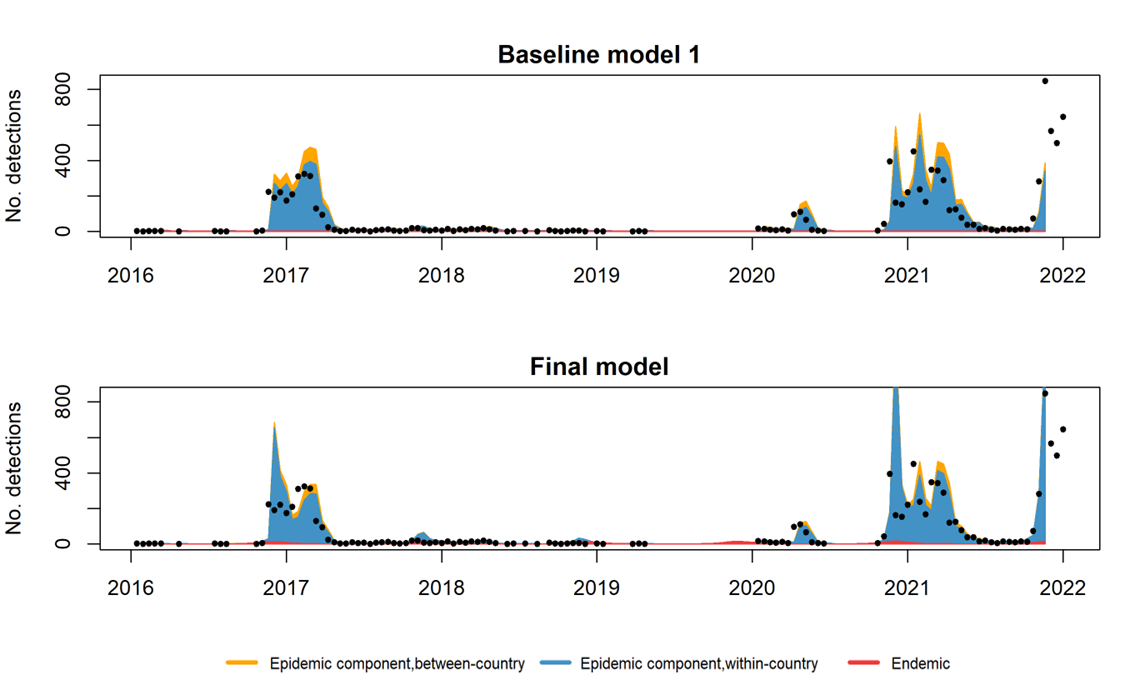
**

**Figure S11.** Overall model fit aggregated over all the 37 countries showing the relative contribution of model components for the biweekly aggregated HPAI1621 baseline model 1 and the final multivariate time-series model. Dots show the actual counts of reported highly pathogenic avian influenza (H5 subtype) detections in domestic and wild birds. Although actual counts from the last biweekly detections of 2021 are depicted, they were not part of the training set in the model, and thus are not part of the model fit.

**Table S1.** HPAI detections and their subtypes reported in the data included in this study, 2016-2022. Number of countries denote the number of countries reporting detected HPAI of the specific subtype.

| **Subtype** | **No. detections** | **No. countries** |
| --- | --- | --- |
| Subtype not specified | 43 | 1 |
| H5 (N not specified) | 214 | 14 |
| H5N1 | 9476 | 35 |
| H5N2 | 10 | 4 |
| H5N3 | 17 | 6 |
| H5N4 | 17 | 4 |
| H5N5 | 124 | 20 |
| H5N6 | 115 | 10 |
| H5N8 | 5572 | 33 |
| H5N9 | 4 | 1 |
| H7N7 | 3 | 2 |
| Total | 15595 |  |

**Table S2.** HPAI H5 subtype detections for the two datasets 2016-2021 and 2021-2022 (from week 39 in 2021 to week 49 in 2022). Note that the two data sets have overlapping data from week 39-52 in 2021.

|  | **2016-2021** | **2021-2022** |
| --- | --- | --- |
| **Subtype** | **No. detections** | **No. detections** |
| H5 (N not specified) | 207 | 29 |
| H5N1 | 3061 | 9305 |
| H5N2 | 7 | 3 |
| H5N3 | 17 | 2 |
| H5N4 | 17 | 0 |
| H5N5 | 107 | 17 |
| H5N6 | 115 | 0 |
| H5N8 | 5565 | 20 |
| H5N9 | 4 | 0 |
| Total | 9100 | 9376 |

**Table S3.** Performance evaluation comparing baseline multivariate time-series models to models including multiple seasonal waves, area of wetland and length of coastline as covariates as well as offsets, and random effects. RPS is the mean ranked probability score and logS is the mean logarithmic score used in the strictly proper scoring rules scheme. HPAI1621 and HPAI2122 are the datasets containing detections from 2016-2021 and 2021-2022 respectively. Calibration test results of p≥0.05 suggest a well-calibrated model. Seasonal waves and covariates were only included in the within-country effects of the epidemic component, whereas power law was added to the between-country effects in the epidemic component. All models included area of country in relation to area of all countries within the study as an offset in the endemic component. The best calibrated models for logS scores are depicted in bold, whereas the second-best models are depicted in bold italic. * depicts significant differences in permutation test between best and second best model (with no significant differences, the second best model could have been chosen as well).

| **Model** | | **HPAI1621** | | | | |
| --- | --- | --- | --- | --- | --- | --- |
| **Endemic** | **Epidemic, within-country** | **RPS** | **Calibration (p-value)** | | **logS** | **Calibration (p-value)** |
| **Seasonality** *(baseline 1 HPAI1621 none\|none]* | | | | | | |
| none | none | 4.188 | 0.065 | | 2.007 | 0.000 |
| *one wave* |  | *4.055* | *0.153* | | 1.953 | *0.061* |
| two waves |  | 4.052 | 0.155 | | 1.955 | 0.066 |
| three waves |  | 4.019 | 0.479 | | 1.949 | 0.011 |
| none | **one wave** | 4.125 | 0.172 | | 2.005 | 0.000 |
| ***one wave*** |  | ***3.997*** | ***0.065*** | | ***1.952*** | ***0.186*** |
| two waves |  | 3.992 | 0.067 | | 1.954 | 0.197 |
| **three waves** |  | **3.959** | **0.250** | | **1.948** | **0.050** |
| none | ***two waves*** | 4.149 | 0.144 | | 2.005 | 0.000 |
| ***one wave*** |  | ***4.024*** | ***0.086*** | | ***1.952*** | ***0.152*** |
| two waves |  | 4.018 | 0.087 | | 1.954 | 0.164 |
| three waves |  | 3.985 | 0.302 | | 1.948 | 0.040 |
| none | three waves | 4.166 | 0.150 | | 2.006 | 0.000 |
| one wave |  | 4.039 | 0.086 | | 1.953 | 0.158 |
| two waves |  | 4.033 | 0.086 | | 1.955 | 0.172 |
| three waves |  | 3.997 | 0.288 | | 1.949 | 0.046 |
| **Covariates and offsets** (*baseline 2 HPAI1621: three waves\|one wave*): | | | | | | |
| ***none*** | ***none*** | ***3.959**** | ***0.250*** | | ***1.948*** | ***0.050*** |
| offset |  | 3.998 | 0.454 | | 1.963 | 0.016 |
| **covariates** |  | **3.956** | **0.217** | | **1.946** | **0.060** |
| none | offset | 4.034 | 0.421 | | 1.957 | 0.014 |
| offset |  | 4.070 | 0.678 | | 1.972 | 0.004 |
| covariates |  | 4.031 | 0.372 | | 1.955 | 0.017 |
| none | covariates | 3.968 | 0.251 | | 1.949 | 0.056 |
| offset |  | 4.008 | 0.452 | | 1.964 | 0.019 |
| covariates |  | 3.964 | 0.218 | | 1.947 | 0.066 |
| **Power law/random effects** *(baseline 3 HPAI1621: covariates\|none)* | | | | | | |
| none | | 3.956 | 0.217 | | 1.946 | 0.060 |
| power law | | 3.920 | 0.005 | | 1.958 | 0.817 |
| random effects (uncorrelated) | | 3.629 | 0.052 | | 1.917 | 0.000 |
| random effects (correlated) | | 3.643 | 0.039 | | 1.915 | 0.000 |
| **power law + random effects (uncorrelated)** | | **3.543** | **0.217** | | **1.882** | **0.648** |
| ***power law + random effects (correlated)*** | | ***3.549*** | ***0.223*** | | ***1.883*** | ***0.630*** |
|  | |  |  | |  |  |
| **Model** | | **HPAI2122** | | | | |
| **Endemic** | **Epidemic, within-country** | **RPS** | | **Calibration (p-value)** | **logS** | **Calibration (p-value)** |
| **Seasonality** *(baseline 1 HPAI2122 none\|none]* | | | | | | |
| none | none | 1.106 | | 0.631 | 1.125 | 0.340 |
| *one wave* |  | 1.090 | | 0.000 | 1.204 | 0.001 |
| two waves |  | 1.100 | | 0.000 | 1.201 | 0.031 |
| three waves |  | 1.100 | | 0.001 | 1.195 | 0.061 |
| none | one wave | 1.074 | | 0.580 | 1.118 | 0.436 |
| one wave |  | 1.057 | | 0.000 | 1.199 | 0.000 |
| two waves |  | 1.065 | | 0.000 | 1.196 | 0.018 |
| three waves |  | 1.064 | | 0.001 | 1.189 | 0.041 |
| **none** | **two waves** | **1.069** | | **0.531** | **1.115** | **0.520** |
| one wave |  | 1.054 | | 0.000 | 1.196 | 0.000 |
| two waves |  | 1.063 | | 0.000 | 1.192 | 0.010 |
| three waves |  | 1.061 | | 0.000 | 1.184 | 0.024 |
| ***none*** | ***three waves*** | ***1.069*** | | ***0.531*** | ***1.115*** | ***0.519*** |
| one wave |  | 1.053 | | 0.000 | 1.197 | 0.000 |
| two waves |  | 1.062 | | 0.000 | 1.193 | 0.012 |
| three waves |  | 1.062 | | 0.001 | 1.184 | 0.028 |
| **Covariates and offsets** *(baseline 2 HPAI2122: none\|two waves)***:** | | | | | | |
| **none** | ***none*** | **1.069** | | **0.531** | **1.115** | **0.520** |
| offset |  | 1.088 | | 0.021 | 1.174 | 0.398 |
| covariates |  | 1.056 | | 0.445 | 1.120 | 0.642 |
| none | offset | 1.159 | | 0.445 | 1.123 | 0.503 |
| offset |  | 1.178 | | 0.013 | 1.183 | 0.378 |
| covariates |  | 1.143 | | 0.401 | 1.126 | 0.587 |
| ***none*** | ***covariates*** | ***1.096*** | | ***0.477*** | ***1.117*** | ***0.538*** |
| offset |  | 1.112 | | 0.016 | 1.176 | 0.368 |
| covariates |  | 1.084 | | 0.417 | 1.122 | 0.636 |
| **Power law/random effects** *baseline 3 HPAI2122 none\|none)***:** | | | | | | |
| *none* | | 1.069 | | 0.531 | 1.115 | 0.520 |
| power law | | 1.042 | | 0.469 | 1.098 | 0.730 |
| random effects (uncorrelated) | | 1.031 | | 0.001 | 1.211 | 0.036 |
| random effects (correlated) | | 1.031 | | 0.001 | 1.208 | 0.049 |
| **power law + random effects (uncorrelated)** | | **1.028** | | **0.306** | **1.091** | **0.738** |
| ***power law + random effects (correlated)*** | | ***1.029*** | | ***0.314*** | ***1.092*** | ***0.714*** |

**Table S4.** Performance evaluation comparing baseline multivariate time-series models of the biweekly aggregated HPAI1621 data set to models including multiple seasonal waves, area of wetland and length of coastline as covariates as well as offsets, and random effects. RPS is the mean ranked probability score and logS is the mean logarithmic score used in the strictly proper scoring rules scheme. Calibration test results of p≥0.05 suggests a well-calibrated model. Seasonal waves and covariates were only included in the within-country effects of the epidemic component, whereas power law was added to the between-country effects in the epidemic component. All models included area of country in relation to area of all countries within the study as an offset in the endemic component. The best calibrated models for logS scores are depicted in bold, whereas the second-best models are depicted in bold italic. There were no significant differences in permutation test between best and second-best models, thus the second-best models could have been chosen as well.

| **Epidemic** | **Epidemic, within-country** | **RPS** | **Calibration (p-value)** | **logS** | **Calibration (p-value)** |
| --- | --- | --- | --- | --- | --- |
| **Seasonality** *(baseline1: none\|none)* | | | | | |
| *none* | *none* | *8.679* | *0.155* | 2.667 | *0.000* |
| *one wave* |  | *8.321* | *0.594* | 2.575 | *0.006* |
| two waves |  | 8.267 | 0.504 | 2.571 | 0.012 |
| three waves |  | 8.219 | 0.846 | 2.578 | 0.004 |
| none | one wave | 10.384 | 0.294 | 2.679 | 0.000 |
| one wave |  | 10.202 | 0.362 | 2.592 | 0.085 |
| two waves |  | 10.132 | 0.300 | 2.589 | 0.124 |
| three waves |  | 10.040 | 0.569 | 2.596 | 0.062 |
| none | ***two waves*** | 10.831 | 0.263 | 2.674 | 0.000 |
| one wave |  | 10.700 | 0.397 | 2.588 | 0.079 |
| ***two waves*** |  | ***10.640*** | ***0.328*** | ***2.585*** | ***0.119*** |
| three waves |  | 10.622 | 0.609 | 2.593 | 0.060 |
| none | **three waves** | 11.495 | 0.224 | 2.674 | 0.000 |
| one wave |  | 11.364 | 0.464 | 2.587 | 0.063 |
| **two waves** |  | **11.258** | **0.387** | **2.584** | **0.095** |
| three waves |  | 11.282 | 0.678 | 2.592 | 0.049 |
| **Covariates and offsets** *(baseline 2: two waves\|three waves)* | | | | | |
| none | ***none*** | 11.258 | 0.387 | 2.584 | 0.095 |
| offset\| none |  | 11.248 | 0.408 | 2.591 | 0.081 |
| ***covariates*** |  | ***11.436*** | ***0.240*** | ***2.572*** | ***0.165*** |
| none | offset | 13.413 | 0.467 | 2.596 | 0.051 |
| offset |  | 13.548 | 0.484 | 2.604 | 0.047 |
| covariates |  | 13.733 | 0.294 | 2.584 | 0.097 |
| *none* | **covariates** | *10.719* | *0.387* | *2.581* | *0.101* |
| offset |  | 10.651 | 0.407 | 2.588 | 0.088 |
| **covariates** |  | **10.832** | **0.238** | **2.568** | **0.172** |
| **Power law/random effects** *(baseline 3: covariates\|covariates)* | | | | | |
| *none* | | *10.832* | *0.238* | *2.568* | *0.172* |
| power law | | 10.688 | 0.029 | 2.589 | 0.889 |
| random effects (uncorrelated) | | 9.710 | 0.000 | 2.596 | 0.000 |
| random effects (correlated) | | 10.173 | 0.000 | 2.606 | 0.000 |
| **power law + random effects (uncorrelated)** | | **9.676** | **0.857** | **2.522** | **0.603** |
| ***power law + random effects (correlated)*** | | ***10.124*** | ***0.937*** | ***2.530*** | ***0.565*** |

**Table S5.** Coefficient estimates from the final multivariate time-series biweekly aggregated HPAI1621 model with 2 seasonal waves in the endemic component, 3 seasonal waves in the within-country effects of the epidemic component, area of country relative to total area of all countries as offset in the endemic component, covariates length of coastline (km) and area of wetlands (km^2^) in the endemic component and within-country effects of the epidemic component, spatial weights *w_ji_* as a power-law model with distance-decay for the between-country effects of the epidemic component and uncorrelated random intercepts for all components.

| **Model components/parameters** | **Coefficient estimate** | **Std. Error** | **2.5% CI** | **97.5% CI** |
| --- | --- | --- | --- | --- |
| ***Endemic component:*** |  |  |  |  |
| Sine(2*π*t/52) | 0.359 | 0.072 | 0.218 | 0.500 |
| Cosine(2*π*t/52) | 2.818 | 0.471 | 1.894 | 3.742 |
| Sine(4*π*t/52) | 0.555 | 0.088 | 0.382 | 0.728 |
| Cosine(4*π*t/52) | 1.046 | 0.185 | 0.684 | 1.409 |
| Length of coastline (km) | -0.10 | 0.145 | -0.380 | 0.186 |
| Area of wetland (km^2^) | 0.172 | 0.313 | -0.442 | 0.785 |
| Random intercept | 0.227 | 0.482 | -0.718 | 1.172 |
| ***Epidemic component:*** |  |  |  |  |
| Sine(2*π*t/52) | 0.629 | 0.095 | 0.443 | 0.815 |
| Cosine(2*π*t/52) | 1.638 | 0.264 | 1.122 | 2.155 |
| Sine(4*π*t/52) | 0.808 | 0.118 | 0.577 | 1.038 |
| Cosine(4*π*t/52) | 0.826 | 0.136 | 0.560 | 1.092 |
| Sine(6*π*t/52) | 1.003 | 0.140 | 0.728 | 1.278 |
| Cosine(6*π*t/52) | 0.720 | 0.102 | 0.520 | 0.920 |
| Length of coastline (km) | -0.091 | 0.034 | -0.158 | -0.025 |
| Area of wetland (km^2^) | 0.052 | 0.068 | -0.082 | 0.186 |
| Within-country random intercept | 0.782 | 0.393 | 0.012 | 1.553 |
| Between country random intercept | 0.110 | 0.024 | 0.062 | 0.158 |
| Spatial weights (*w_ji_*) | 17.007 | 5.713 | 5.811 | 28.204 |

**References**

1. Natural Earth. Admin 0 – Countries - Free vector and raster map data at 1:10m, 1:50m, and 1:110m scales. https://www.naturalearthdata.com/downloads/10m-cultural-vectors/10m-admin-0-countries/ (2021).

2. Spina, F., Baillie, S. R., Bairlein, F., Fiedler, W. & Thorup, K. The Eurasian African Bird Migration Atlas. EURING/CMS https://migrationatlas.org/ (2022).

3. Tennekes, M. tmap: Thematic Maps in R. J. Stat. Softw. 84, 1-39 (2018).

4. R Development Core Team. R: A Language and Environment for Statistical Computing. R Foundation for Statistical Computing http://www.r-project.org Preprint at http://www.r-project.org (2022).

5. Meyer, S., Held, L. & Höhle, M. Spatio-Temporal Analysis of Epidemic Phenomena Using the R Package surveillance. J Stat Softw 77, (2014).

6. Salmon, M., Schumacher, D. & Höhle, M. Monitoring Count Time Series in R: Aberration Detection in Public Health Surveillance. J Stat Softw 70, 1–35 (2016).
